# The association between SGLT2 inhibitors and rheumatoid arthritis risk in type 2 diabetes: findings from large-scale emulated target trials

**DOI:** 10.3389/fimmu.2026.1867117

**Published:** 2026-08-04

**Authors:** Fu-Shun Yen, Shiow-Ing Wang, Chih-Cheng Hsu, Sung Huang Laurent Tsai, Chii-Min Hwu, James Cheng-Chung Wei

**Affiliations:** 1 Dr. Yen’s Clinic, Taoyuan, Taiwan; 2 Center for Health Data Science, Department of Medical Research, Chung Shan Medical University Hospital, Taichung, Taiwan; 3 Department of Health Policy and Management, College of Health Care and Management, Chung Shan Medical University, Taichung, Taiwan; 4 Institute of Population Health Sciences, National Health Research Institutes, Miaoli, Taiwan; 5 Department of Health Services Administration, China Medical University, Taichung, Taiwan; 6 Department of Family Medicine, Min-Sheng General Hospital, Taoyuan, Taiwan; 7 National Center for Geriatrics and Welfare Research, National Health Research Institutes, Huwei, Yunlin, Taiwan; 8 Department of Orthopedics, Taipei Medical University Hospital, Taipei, Taiwan; 9 Department of Orthopaedics, School of Medicine, College of Medicine, Taipei Medical University, Taipei, Taiwan; 10 Department of Biomedical Engineering, National Taiwan University, Taipei, Taiwan; 11 Graduate Institute of Clinical Medicine, College of Medicine, Taipei Medical University, Taipei, Taiwan; 12 Section of Endocrinology and Metabolism, Department of Medicine, Taipei Veterans General Hospital, Taipei, Taiwan; 13 Faculty of Medicine, National Yang Ming Chiao Tung University School of Medicine, Taipei, Taiwan; 14 Department of Allergy, Immunology & Rheumatology, Chung Shan Medical University Hospital, Taichung, Taiwan; 15 Graduate Institute of Integrated Medicine, China Medical University, Taichung, Taiwan

**Keywords:** dipeptidyl peptidase-4 inhibitors, pioglitazone, rheumatoid arthritis, sodium-glucose cotransporter-2 inhibitors, sulfonylureas

## Abstract

**Introduction:**

Globally, the prevalence of rheumatoid arthritis (RA) is on the rise. Certain antidiabetic medications have been reported to lower the risk of RA. In this study, we conducted multicenter emulated target trials to compare the risk of RA development between sodium-glucose cotransporter-2 inhibitors (SGLT2i) and non-SGLT2i treatment options in patients with type 2 diabetes mellitus (T2DM).

**Methods:**

We identified 4,991,988 patients with T2DM from the TriNetX network between January 1, 2016, to June 30, 2023. From this cohort, we selected 310,507, 206,069, and 80,846 propensity score-matched pairs of patients using SGLT2i versus sulfonylureas, dipeptidyl peptidase-4 inhibitors (DPP-4i), and pioglitazone, respectively. The Kaplan-Meier method was used to determine the relative hazards of developing RA among these groups. Subgroup analyses were conducted based on sex, age, race, obesity status, HbA1C levels, and eGFR, with an additional sensitivity analysis using the intention-to-treat approach.

**Results:**

SGLT2i use was associated with a significantly lower risk of incident RA compared to sulfonylurea use (HR: 0.899, 95% CI: 0.835–0.968). However, there was no significant difference in RA risk when comparing SGLT2i with DPP-4 inhibitors (HR: 0.914, 95% CI: 0.831–1.005) or pioglitazone (HR: 0.922, 95% CI: 0.796–1.068). Kaplan-Meier curves demonstrated that SGLT2i users had a significantly lower likelihood of developing RA than sulfonylurea users (log-rank p = 0.004). Subgroup analyses further indicated that SGLT2i use was consistently associated with a lower risk of RA across all analyzed patient subgroups compared to sulfonylureas. Additionally, sensitivity analyses confirmed the robustness of these findings, aligning with the results from the emulated target trial comparing SGLT2i to sulfonylureas.

**Discussion:**

This study found that SGLT2i use was associated with a lower risk of incident RA compared with sulfonylurea use among patients with T2DM. However, because of the observational study design, these findings should be interpreted as associations rather than evidence of causality.

## 1 Introduction

Rheumatoid arthritis (RA) is the most common chronic inflammatory disease worldwide, often leading to musculoskeletal impairment, disability, and premature death (1, 2).Between 1990 and 2020, the global age-standardized prevalence rate rose by approximately 14.1% (3). In the United States, the prevalence of RA is estimated to be between 0.5% and 1%, affecting over 1.3 million adults. The annual incidence is around 40 per 100, 000 individuals, with age- and sex-adjusted rates of 41 per 100, 000 (53 per 100, 000 in women and 29 per 100, 000 in men) between 2005 and 2014 (4). Clearly, the prevalence of RA has been increasing globally.

RA may contribute to insulin resistance and type 2 diabetes mellitus (T2DM) due to the frequent use of corticosteroids and high levels of systemic inflammation (5, 6). Conversely, individuals with T2DM, may have an increased risk of RA, possibly due to obesity-related metabolic syndrome and chronic low-grade inflammation (7). Some antidiabetic medications have been reported to reduce the risk of RA through their effects on improving insulin resistance, lowering blood glucose levels, and reducing inflammation (8–12). A nationwide cohort study found that sulfonylureas and biguanides were associated with a lower risk of developing RA than non-sulfonylurea and non-biguanide treatments in patients with T2DM (8). Moreover, a systematic review and meta-analysis of ten clinical trials concluded that dipeptidyl peptidase-4 inhibitors (DPP-4i) do not increase the risk of RA (9). However, another meta-analysis of four cohort studies found that the use of DPP-4i was associated with a 28% lower risk of developing RA compared with non-use (10). A retrospective cohort study demonstrated that patients using thiazolidinediones (TZDs) had a lower risk of developing RA compared to those using alpha glucosidase inhibitors (AGIs) (11). Additionally, a case-control study indicated that among patients with T2DM, those taking TZDs showed a trend toward reduced rheumatoid arthritis risk compared to non-TZD users, though this reduction did not reach statistical significance (12).

With respect to newer glucose-lowering therapies, evidence regarding the association between sodium–glucose cotransporter-2 inhibitors (SGLT2is) and glucagon-like peptide-1 receptor agonists (GLP-1 RAs) and the risk of RA remains limited. SGLT2is exert their glucose-lowering effect by enhancing renal glucose excretion. Beyond glycemic control, these agents have been shown to attenuate oxidative stress and exert anti-inflammatory properties.¹³ Experimental studies suggest additional immunometabolic effects of SGLT2is, including enhanced lipid utilization, increased ketone body production, suppression of NLRP3 inflammasome activation, and reduced secretion of interleukin (IL)-1β.¹^4^ Canagliflozin has been demonstrated to inhibit T-cell activation and proliferation through downregulation of mammalian target of rapamycin complex 1 (mTORC1) in CD4^+^ T cells isolated from patients with systemic lupus erythematosus and RA (15). Furthermore, experiments using synovial fluid mononuclear cells from patients with RA have shown that canagliflozin reduces IL-17 expression (15, 16). Despite these mechanistic insights, clinical evidence evaluating the impact of SGLT2i on the incidence of RA is still scarce. We therefore hypothesized that initiation of SGLT2i therapy would be associated with a lower incidence of RA. we conducted multicenter emulated target trials (17) to compare the incidence of RA among patients with T2DM receiving SGLT2is versus those treated with alternative glucose-lowering agents.

## 2 Patients and methods

### 2.1 Study design and data source

This study was conducted as a retrospective cohort analysis using data from TriNetX, the world’s largest real-world data and evidence ecosystem in the life sciences and healthcare sector. The platform has been widely used for high-quality research (18, 19).

TriNetX integrates de-identified electronic health records from over 250 million individuals across more than 120 global healthcare organizations (HCOs). These records undergo processing through data warehouses and research repositories before being incorporated into the TriNetX platform. The dataset includes structured information, such as demographics, diagnoses, procedures, medications, laboratory test results, and vital signs. TriNetX captures both inpatient and outpatient data, with most HCOs contributing comprehensive datasets. To ensure data quality, TriNetX employs a standardized framework that evaluates three key metrics: conformance, completeness, and plausibility (20).

The data and analysis for this study were conducted in January 2025. We utilized the US Collaborative Network, a subset of the TriNetX platform, which included 69 healthcare organizations. In accordance with our study objectives, we restricted the study period to data collected between January 1, 2016, and December 31, 2024.

### 2.2 Ethics statement

The TriNetX platform complies with the Health Insurance Portability and Accountability Act (HIPAA) and the General Data Protection Regulation (GDPR). The Western Institutional Review Board (WIRB) has granted TriNetX a waiver, as the database provides aggregated counts and statistical summaries of de-identified data. Additionally, the use of TriNetX for this study was approved by the Institutional Review Board of Chung Shan Medical University Hospital (CSMUH Approval No: CS2-21176).

### 2.3 Target trials

We applied the framework proposed by Hernán and Robins (2016) (15) to emulate clinical trials comparing SGLT2i treatment with non-SGLT2i treatment (DPP-4i, pioglitazone, or sulfonylureas) in patients with T2DM using an unblinded design. First, we established the target trial emulation framework to align with our research objectives, incorporating key elements, such as eligibility criteria, treatment strategies, allocation methods, follow-up procedures, outcomes of interest, causal comparisons, and the analysis plan (**Supplementary Table 1**) (21). Next, we defined the approach for leveraging observational data to replicate these protocol components and conduct the corresponding analyses (**Supplementary Table 2**).

### 2.4 Study eligibility criteria

Individuals eligible for this study were those with at least two documented diagnoses of T2DM, identified using the International Statistical Classification of Diseases, Tenth Revision, Clinical Modification (ICD-10-CM) codes (**Supplementary Table 3**). We excluded individuals under 20 years of age and those with a history of type 1 diabetes mellitus. Additionally, to focus on recently diagnosed cases and assess treatment outcomes in patients with newly diagnosed T2DM, we excluded individuals diagnosed with T2DM before December 31, 2015. Furthermore, patients were excluded from both cohorts if they had a history of dialysis or kidney transplantation, as these conditions could introduce confounding factors related to comorbidities that might influence the study outcomes.

### 2.5 Assigned treatment strategies and groups

In the first target trial, individuals classified as SGLT2i users were those who received an SGLT2i prescription following their initial diagnosis of T2DM. SGLT2i use was identified using the Anatomical Therapeutic Chemical (ATC) code A10BK, with the index date defined as the date of the first SGLT2i prescription. Similarly, the sulfonylurea cohort consisted of individuals who were prescribed sulfonylureas (ATC code A10BB) after their first T2DM diagnosis, with the index date set as the date of their first sulfonylurea prescription. The second target trial compared the initiation of SGLT2i to DPP-4i at baseline, while the third target trial compared the initiation of SGLT2i to pioglitazone at baseline. In all scenarios, the initiation date (index date) was defined as the date of filling the first prescription for the respective medication. Each emulated target trial was constructed independently from the original eligible T2DM source population. Patients were re-selected and propensity score matched separately for each treatment comparison. Therefore, treatment assignment was specific to each target trial and was determined solely by the index medication. Within each comparison, the two comparator-defining medications were mutually exclusive at baseline, whereas other glucose-lowering medications could be present as baseline comedications and were included as covariates in the propensity score matching. To preserve the intention-to-treat design, individuals were not excluded if they switched treatment during the study period. However, patients in both cohorts were excluded if they had a prior diagnosis of RA, or had died on or before the index date.

### 2.6 Study outcomes and follow-up

The primary outcome was incident rheumatoid arthritis, identified using ICD-10-CM codes M05 and M06 recorded after the index date. Individuals with any diagnosis of RA before cohort entry were excluded to improve the specificity of incident case ascertainment. The secondary outcomes included all-cause mortality, and medical utilization (defined as hospital inpatient services, emergency department visits, and critical care services). To assess the risk of the specified outcomes, each participant was followed from the initiation of the assigned treatment, for up to one year.

### 2.7 Emulation of target trials

To simulate randomization, propensity score matching (PSM) was employed to estimate the probability (propensity score) of treatment assignment (e.g., initiation of SGLT2i, sulfonylureas, DPP-4i, or pioglitazone) based on observed baseline characteristics. SGLT2i initiators were matched 1:1 with contemporaneous initiators of sulfonylureas, DPP-4i, or pioglitazone. The matching process utilized the TriNetX propensity score generation tool, applying 1:1 greedy nearest neighbor matching with a caliper width of 0.1 pooled standard deviations for the matching variables. The balance between cohorts, before and after matching, was assessed using standardized mean differences (SMDs), with SMDs below 0.1 indicating well-balanced groups.

To account for potential confounding, variables were analyzed during the one year prior to the index date. The variables were included as follows. 1. Demographic and socioeconomic factors (age at the index date, sex, race, and socioeconomic status). 2. Lifestyle factors, such as smoking status (tobacco use, nicotine dependence, personal history of nicotine dependence, and alcohol-related disorders). 3. Diabetes severity indicators (T2DM complications including hyperosmolarity, ketoacidosis, kidney complications, ophthalmic complications, neurological complications, circulatory complications, other specified complications, unspecified complications, and T2DM without complications). 4. Medical utilization (office or other outpatient services, emergency services, inpatient services, and preventive care services). 5. Comorbidities (defined by ICD-10 Codes and categorized as present or absent) including hypertensive diseases, ischemic heart disease, heart failure, cerebrovascular diseases, diseases of the arteries, arterioles, and capillaries, overweight and obesity, dyslipidemia, chronic kidney disease, unspecified kidney failure, unspecified proteinuria, chronic lower respiratory diseases, depressive episodes, recurrent major depressive disorder, liver diseases, viral hepatitis, psoriasis, systemic lupus erythematosus, and ankylosing spondylitis. 6. Medication use, such as metformin, thiazolidinediones, DPP-4i, GLP-1 RAs, insulin and analogs, statins, beta-blockers, diuretics, angiotensin-converting enzyme (ACE) inhibitors, angiotensin II receptor blockers, calcium channel blockers, aspirin, corticosteroids for systemic use, proton pump inhibitors, non-steroidal anti-inflammatory drugs (NSAIDs), and alpha-adrenoreceptor antagonists. 7. Laboratory data: hemoglobin A1C (HbA1c), estimated glomerular filtration rate (eGFR; calculated using the Modification of Diet in Renal Disease [MDRD] formula), body mass index (BMI), and rheumatoid factor levels. By integrating these variables into the propensity score matching process, we aimed to reduce confounding and ensure comparability between treatment groups, thus, enhancing the validity of our target trial emulation.

### 2.8 Analysis plan and statistical methods

The primary causal estimands in this study were the intention-to-treat (ITT) effects of the assigned treatment strategies. To estimate hazard ratios (HRs) and confidence intervals (CIs) and assess the proportionality of outcome risks, we utilized the Survival package (version 3.2-3) in R. The proportional hazards assumption was assessed using the proportionality test provided within the TriNetX platform together with visual inspection of Kaplan–Meier survival curves.

Kaplan-Meier analysis was performed to estimate the cumulative probability of developing RA within the emulated target trials. Differences between survival curves for the treatment groups were assessed using the log-rank test. To explore variations in RA risk, we conducted the following subgroup analyses: sex (male, female); age groups (20–44 years, 45–64 years, ≥65 years); race (White, African-American, Asian); obesity status (with and without obesity); HbA1c levels (<7%, ≥9%); and eGFR categories (<60, 60-90, ≥90 ml/min/1.73 m²).

To assess the robustness of our findings, we performed additional sensitivity analyses. First, using different sets of matching variables while maintaining the same study design. Second, adjusting the study design by excluding participants who switched treatments during follow-up. Third, conducting the same study design within a different TriNetX network to evaluate consistency across datasets. Fourth, we performed additional analyses using longer maximum follow-up durations of 3, 5, and 7 years.

## 3 Results

### 3.1 Characteristics of study subjects

**Figure 1:**
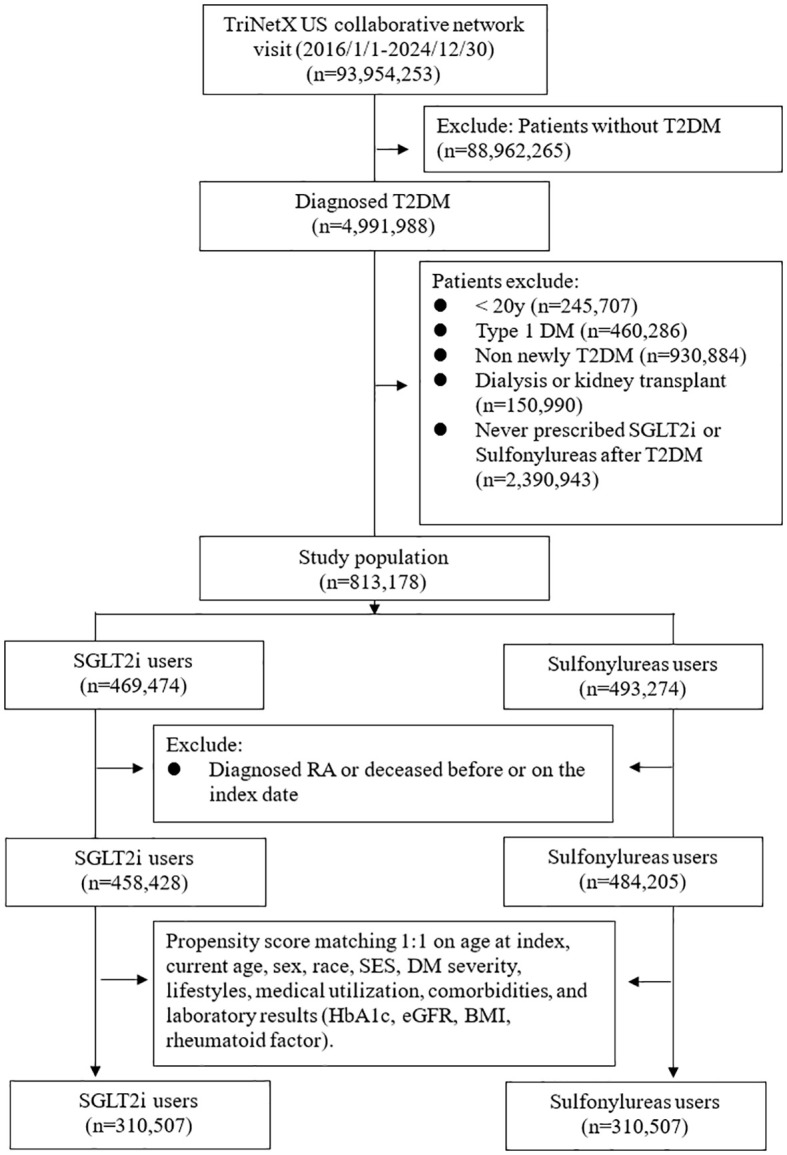
Selection process (SGLT2i vs. sulfonylureas). T2DM, type 2 diabetes mellitus; SGLT2i: Sodium-glucose Cotransporter-2 Inhibitors; SES, socioeconomic status; HbA1c, hemoglobin A1C; eGFR, estimated glomerular filtration rate; BMI, body mass index.

In this study, we initially identified 4, 991, 988 patients with T2DM. After excluding ineligible individuals, the cohort included 458, 428 users of SGLT2i and 484, 205 users of sulfonylureas. Following propensity score matching (PSM), 310, 507 patients were classified as SGLT2i users, with an equal number in the sulfonylurea group (**Figure 1**). Baseline characteristics of the study population, before and after matching, are presented in **Table 1**. Prior to matching, significant differences were observed between SGLT2i and sulfonylurea users in terms of age, diabetic kidney complications, healthcare utilization, comorbidities, concomitant medications, and laboratory findings. After PSM, covariate balance was substantially improved. Most baseline characteristics achieved an SMD <0.1; however, residual imbalance remained for GLP-1 RA use and angiotensin II receptor blocker use. The follow-up duration for the target trial comparing SGLT2i and sulfonylureas was similar between the two groups. The median follow-up time was 365 days (interquartile range [IQR], 10) for SGLT2i users and 365 days (IQR, 67) for sulfonylurea users.

**Table 1:** Baseline characteristics of study participants: SGLT2i vs. Sulfonylureas (before and after matching).

Variables	Before PSM			After PSM^a^		
	SGLT2i users (n=458428)	Sulfonylureas users (n=484205)	SMD	SGLT2i users (n=310507)	Sulfonylureas users (n=310507)	SMD
Current age, mean ± SD	63.9 ± 12.2	66.3 ± 12.9	**0.188**	64.6 ± 11.9	64.4 ± 12.9	0.014
Age at index, mean ± SD	61.0 ± 12.4	61.7 ± 12.8	0.052	61.0 ± 12.2	60.8 ± 13.0	0.015
Sex, n (%)						
Male	256391 (55.9)	256674 (53.0)	0.059	168794 (54.4)	169553 (54.6)	0.005
Female	188818 (41.2)	216804 (44.8)	0.073	133754 (43.1)	133082 (42.9)	0.004
Unknown Gender	13219 (2.9)	10727 (2.2)	0.042	7959 (2.6)	7872 (2.5)	0.002
Race, n (%)						
White	268537 (58.6)	289629 (59.8)	0.025	183123 (59.0)	184483 (59.4)	0.009
Black or African American	71272 (15.5)	76519 (15.8)	0.007	47598 (15.3)	46726 (15.0)	0.008
Unknown Race	60768 (13.3)	63071 (13.0)	0.007	41571 (13.4)	41367 (13.3)	0.002
Other Race	26623 (5.8)	27094 (5.6)	0.009	18482 (6.0)	18417 (5.9)	0.001
Asian	26786 (5.8)	23929 (4.9)	0.040	16976 (5.5)	16924 (5.5)	0.001
Native Hawaiian or Other Pacific Islander	2754 (0.6)	2343 (0.5)	0.016	1635 (0.5)	1558 (0.5)	0.003
American Indian or Alaska Native	1688 (0.4)	1620 (0.3)	0.006	1122 (0.4)	1032 (0.3)	0.005
Social economic status, n (%)						
Persons with potential health hazards related to socioeconomic and psychosocial circumstances	10223 (2.2)	7707 (1.6)	0.047	5695 (1.8)	5459 (1.8)	0.006
Lifestyles, n (%)						
Personal history of nicotine dependence	50327 (11.0)	42789 (8.8)	0.072	29263 (9.4)	28210 (9.1)	0.012
Nicotine dependence	42664 (9.3)	41177 (8.5)	0.028	26665 (8.6)	25632 (8.3)	0.012
Tobacco use	14980 (3.3)	12198 (2.5)	0.045	8536 (2.7)	8300 (2.7)	0.005
Alcohol related disorders	9118 (2.0)	8814 (1.8)	0.012	5600 (1.8)	5262 (1.7)	0.008
DM severity, n (%)						
Type 2 diabetes mellitus without complications	287429 (62.7)	287608 (59.4)	0.068	189956 (61.2)	186177 (60.0)	0.025
Type 2 diabetes mellitus with other specified complications	166242 (36.3)	147669 (30.5)	**0.123**	106794 (34.4)	104427 (33.6)	0.016
Type 2 diabetes mellitus with kidney complications	64526 (14.1)	44314 (9.2)	**0.154**	33636 (10.8)	32649 (10.5)	0.010
Type 2 diabetes mellitus with neurological complications	51791 (11.3)	42640 (8.8)	0.083	31071 (10.0)	30263 (9.7)	0.009
Type 2 diabetes mellitus with circulatory complications	30881 (6.7)	21646 (4.5)	0.099	17050 (5.5)	16506 (5.3)	0.008
Type 2 diabetes mellitus with unspecified complications	19075 (4.2)	18761 (3.9)	0.015	12422 (4.0)	11851 (3.8)	0.009
Type 2 diabetes mellitus with ophthalmic complications	18713 (4.1)	12674 (2.6)	0.081	10017 (3.2)	9792 (3.2)	0.004
Type 2 diabetes mellitus with hyperosmolarity	4863 (1.1)	4517 (0.9)	0.013	3122 (1.0)	3020 (1.0)	0.003
Type 2 diabetes mellitus with ketoacidosis	3521 (0.8)	3605 (0.7)	0.003	2512 (0.8)	2411 (0.8)	0.004
Medical utilization, n (%)						
Office or other outpatient services	239503 (52.2)	201064 (41.5)	**0.216**	147533 (47.5)	145152 (46.7)	0.015
Emergency department services	94227 (20.6)	102021 (21.1)	0.013	61695 (19.9)	59151 (19.1)	0.021
Hospital inpatient and observation care services	70639 (15.4)	59188 (12.2)	0.092	39705 (12.8)	38540 (12.4)	0.011
Preventive medicine services	39000 (8.5)	28369 (5.9)	**0.103**	22916 (7.4)	22179 (7.1)	0.009
Comorbidities, n (%)						
Hypertensive diseases	304896 (66.5)	286779 (59.2)	**0.151**	193048 (62.2)	188598 (60.7)	0.029
Disorders of lipoprotein metabolism and other lipidemias	279171 (60.9)	236586 (48.9)	**0.244**	171456 (55.2)	168661 (54.3)	0.018
Overweight and obesity	129383 (28.2)	101506 (21.0)	**0.169**	76070 (24.5)	74209 (23.9)	0.014
Ischemic heart diseases	111010 (24.2)	77029 (15.9)	**0.209**	57248 (18.4)	55931 (18.0)	0.011
Neoplasms	62982 (13.7)	59393 (12.3)	0.044	40027 (12.9)	38892 (12.5)	0.011
Chronic lower respiratory diseases	66731 (14.6)	59801 (12.4)	0.065	40194 (12.9)	38604 (12.4)	0.015
Chronic kidney disease	68896 (15.0)	48464 (10.0)	**0.152**	35903 (11.6)	34579 (11.1)	0.013
Heart failure	83007 (18.1)	40477 (8.4)	**0.291**	33459 (10.8)	33132 (10.7)	0.003
Depressive episode	45141 (9.8)	40389 (8.3)	0.052	27920 (9.0)	27049 (8.7)	0.010
Diseases of arteries, arterioles and capillaries	43510 (9.5)	30729 (6.3)	**0.117**	22464 (7.2)	22093 (7.1)	0.005
Cerebrovascular diseases	34172 (7.5)	31226 (6.4)	0.040	20481 (6.6)	19721 (6.4)	0.010
Diseases of liver	34818 (7.6)	27919 (5.8)	0.073	20473 (6.6)	19931 (6.4)	0.007
Proteinuria, unspecified	21710 (4.7)	10879 (2.2)	**0.136**	9612 (3.1)	9281 (3.0)	0.006
Major depressive disorder, recurrent	13707 (3.0)	10037 (2.1)	0.058	7894 (2.5)	7671 (2.5)	0.005
Psoriasis	4726 (1.0)	3927 (0.8)	0.023	2847 (0.9)	2826 (0.9)	0.001
Viral hepatitis	4346 (0.9)	4606 (1.0)	0.000	2705 (0.9)	2621 (0.8)	0.003
Systemic lupus erythematosus	1075 (0.2)	826 (0.2)	0.014	623 (0.2)	574 (0.2)	0.004
Unspecified kidney failure	766 (0.2)	948 (0.2)	0.007	523 (0.2)	475 (0.2)	0.004
Ankylosing spondylitis	413 (0.1)	325 (0.1)	0.008	237 (0.1)	221 (0.1)	0.002
Comedication, n (%)						
Metformin	251688 (54.9)	291684 (60.2)	**0.108**	179121 (57.7)	187959 (60.5)	0.058
Glucagon-like peptide-1 analogues	92147 (20.1)	46875 (9.7)	**0.296**	61718 (19.9)	38035 (12.2)	**0.209**
Dipeptidyl peptidase 4 inhibitors	61689 (13.5)	67677 (14.0)	0.015	47866 (15.4)	43487 (14.0)	0.040
Thiazolidinediones	19224 (4.2)	23756 (4.9)	0.034	14388 (4.6)	15653 (5.0)	0.019
Insulins and analogues	167785 (36.6)	157366 (32.5)	0.086	110272 (35.5)	98581 (31.7)	0.080
HMG CoA reductase inhibitors	288654 (63.0)	276456 (57.1)	**0.120**	190582 (61.4)	182024 (58.6)	0.056
Beta blocking agents	180210 (39.3)	170170 (35.1)	0.086	110559 (35.6)	108001 (34.8)	0.017
Diuretics	177852 (38.8)	164058 (33.9)	**0.102**	109021 (35.1)	104698 (33.7)	0.029
ACE inhibitors	137346 (30.0)	161622 (33.4)	0.074	95302 (30.7)	99380 (32.0)	0.028
Corticosteroids for systemic use	130784 (28.5)	124083 (25.6)	0.065	84204 (27.1)	82052 (26.4)	0.016
Angiotensin II receptor blockers	138455 (30.2)	105256 (21.7)	**0.194**	86080 (27.7)	70494 (22.7)	**0.116**
Proton pump inhibitors	120330 (26.2)	120528 (24.9)	0.031	78503 (25.3)	77273 (24.9)	0.009
Aspirin	120656 (26.3)	123672 (25.5)	0.018	75715 (24.4)	74598 (24.0)	0.008
Calcium channel blockers	120570 (26.3)	120839 (25.0)	0.031	76909 (24.8)	77138 (24.8)	0.002
NSAIDs	105291 (23.0)	110747 (22.9)	0.002	72384 (23.3)	70781 (22.8)	0.012
Alpha-adrenoreceptor antagonists	5348 (1.2)	6430 (1.3)	0.015	3369 (1.1)	4098 (1.3)	0.022
Laboratory, n (%)						
Hemoglobin A1c/Hemoglobin. total in Blood (%)						
< 7	107387 (23.4)	76140 (15.7)	**0.195**	57546 (18.5)	56572 (18.2)	0.008
≧9	93956 (20.5)	87610 (18.1)	0.061	63592 (20.5)	61655 (19.9)	0.016
Estimated Glomerular filtration rate (eGFR^b^, ml/min/1.73m^2^)						
< 60	116209 (25.3)	100623 (20.8)	**0.109**	68316 (22.0)	66031 (21.3)	0.018
60 ~ 90	184008 (40.1)	159560 (33.0)	**0.150**	113050 (36.4)	110767 (35.7)	0.015
≧90	135913 (29.6)	126706 (26.2)	0.078	88232 (28.4)	86596 (27.9)	0.012
Body mass index (BMI, kg/m2)						
≧30	206508 (45.0)	184521 (38.1)	**0.141**	129658 (41.8)	127524 (41.1)	0.014
Rheumatoid factor [units/volume] in serum or plasma (IU/mL)						
≧30	151 (0.0)	124 (0.0)	0.004	83 (0.0)	84 (0.0)	0.000

Bold font represents a standardized difference was more than 0.1.

If the patient is less or equal to 10, results show the count as 10.

PSM, Propensity score matching, SGLT2i, Sodium-glucose Cotransporter-2 Inhibitors, SMD, Standardized mean difference, SD, Standard deviation, DM, Diabetes mellitus, ACE, Angiotensin-converting enzyme, HMG CoA, β-Hydroxy β-methylglutaryl-CoA, NSAIDs, Anti-inflammatory and anti-rheumatic products, non-steroids.

^a^Propensity score matching was performed on age at index, current age, sex, race, socioeconomic status, DM severity, lifestyles, medical utilization, comorbidities, and HbA1C, eGFR, BMI, rheumatoid factor.

^b^Predicted by Creatinine-based formula (modification of diet in renal disease, MDRD).

Similarly, we applied the same methodology to construct an emulated target trial comparing SGLT2i with DPP-4i using 1:1 PSM, resulting in 206, 069 matched pairs (**Supplementary Figure 1**). Most baseline characteristics achieved an SMD <0.1, although residual imbalance remained for GLP-1 RA use and sulfonylurea use (**Supplementary Table 4**). Additionally, we conducted an emulated target trial comparing SGLT2i with pioglitazone, yielding 80, 846 matched pairs (**Supplementary Figure 2**). In this comparison, most baseline characteristics achieved an SMD <0.1, although residual imbalance remained for sulfonylurea use (**Supplementary Table 5**).

### 3.2 Main outcomes

#### 3.2.1 SGLT2i users versus sulfonylurea users

**Figure 2:**
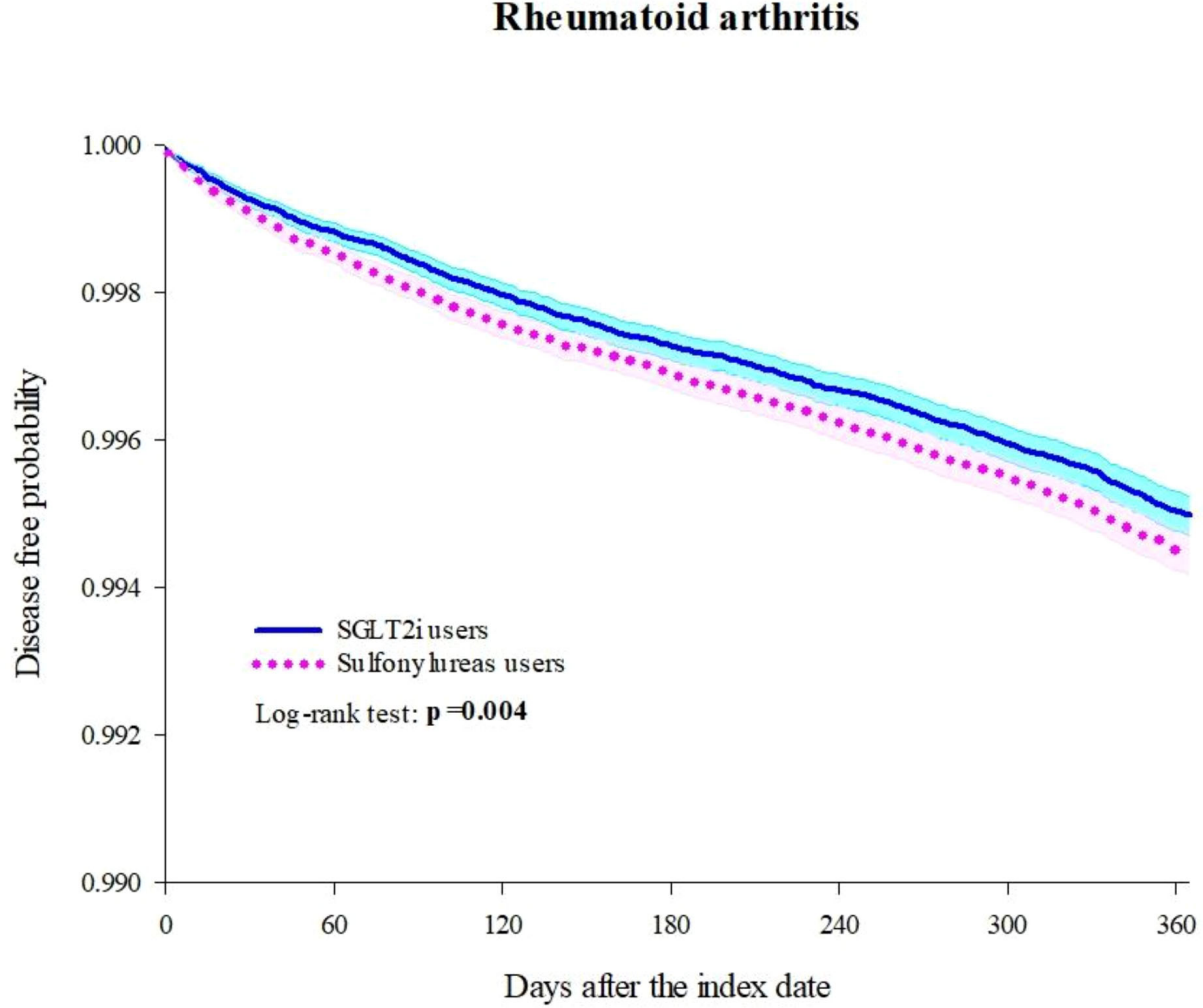
Kaplan-Meier curve for rheumatoid arthritis.

In the emulated target trial comparing SGLT2i with sulfonylureas, the cumulative absolute risk of incident RA during the one-year follow-up was 0.118% among SGLT2i users and 0.131% among sulfonylurea users. Correspondingly, SGLT2i use was associated with a significantly lower risk of incident RA (HR, 0.899; 95% CI, 0.835–0.968; **Table 2**). The Kaplan-Meier curves illustrating RA incidence in both groups are presented in **Figure 2**. The log-rank test indicated a significant difference in RA incidence between the two cohorts (p = 0.004). Additionally, in this emulated target trial, SGLT2i users demonstrated significantly lower healthcare utilization and a reduced risk of all-cause mortality compared to sulfonylurea users (**Table 2**).

**Table 2:** Rheumatoid arthritis risks (1 day to 1 year).

Outcome	One-year cumulative incidence				Hazard ratio (95%CI) ^a^	Risk difference (95%CI)
	SGLT2i use (n=310, 507)	Absolute risk estimates	Sulfonylurea use (n=310, 507)	Absolute risk estimates		
Rheumatoid arthritis	1343	0.43%	1454	0.47%	**0.899** **(0.835-0.968)**	-0.0004 (-0.0007~ -0.00002)
Medical utilization						
Hospitalization	63288	20.4%	74765	24.1%	**0.815** (0.806-0.824)^*^	-0.037 (-0.039~ -0.035)
Emergency room visit	46932	15.1%	52099	16.8%	**0.869** **(0.859-0.880)**	-0.017 (-0.018~ -0.015)
Critical care	10125	3.26%	11674	3.76%	**0.847** (0.825-0.870)^*^	-0.005 (-0.006~ -0.004)
All-cause mortality						
Deceased	5606	1.81%	7381	2.38%	**0.740** **(0.715-0.766)**	-0.006 (-0.006~ -0.005)

SGLT2i, Sodium-glucose cotransporter-2 inhibitors; CI, Confidence interval.

If the patient number is less or equal to 10, results show the count as 10.

^a^Propensity score matching was performed on age at index, sex, race, socioeconomic status, DM severity, lifestyles, medical utilization, comorbidities, and HbA1C, eGFR, BMI, and rheumatoid factor.

^*^Proportionality <0.001.

Bold values indicate statistically significant associations.

#### 3.2.2 SGLT2i users versus DPP-4i users

In the emulated target trial comparing SGLT2i with DPP-4i, SGLT2i use was not associated with a significant difference in the risk of RA (HR: 0.914, 95% CI: 0.831–1.005). However, SGLT2i use was significantly linked to a lower risk of hospitalization, emergency room visits, and all-cause mortality (**Supplementary Table 6**).

#### 3.2.3 SGLT2i users versus pioglitazone users

In the emulated target trial comparing SGLT2i with pioglitazone, SGLT2i use was not significantly associated with the risk of RA (HR: 0.922, 95% CI: 0.796–1.068). However, SGLT2i use was significantly associated with lower healthcare utilization and a reduced risk of all-cause mortality (**Supplementary Table 7**).

### 3.3 Subgroup analyses: SGLT2i vs. sulfonylureas

**Figure 3:**
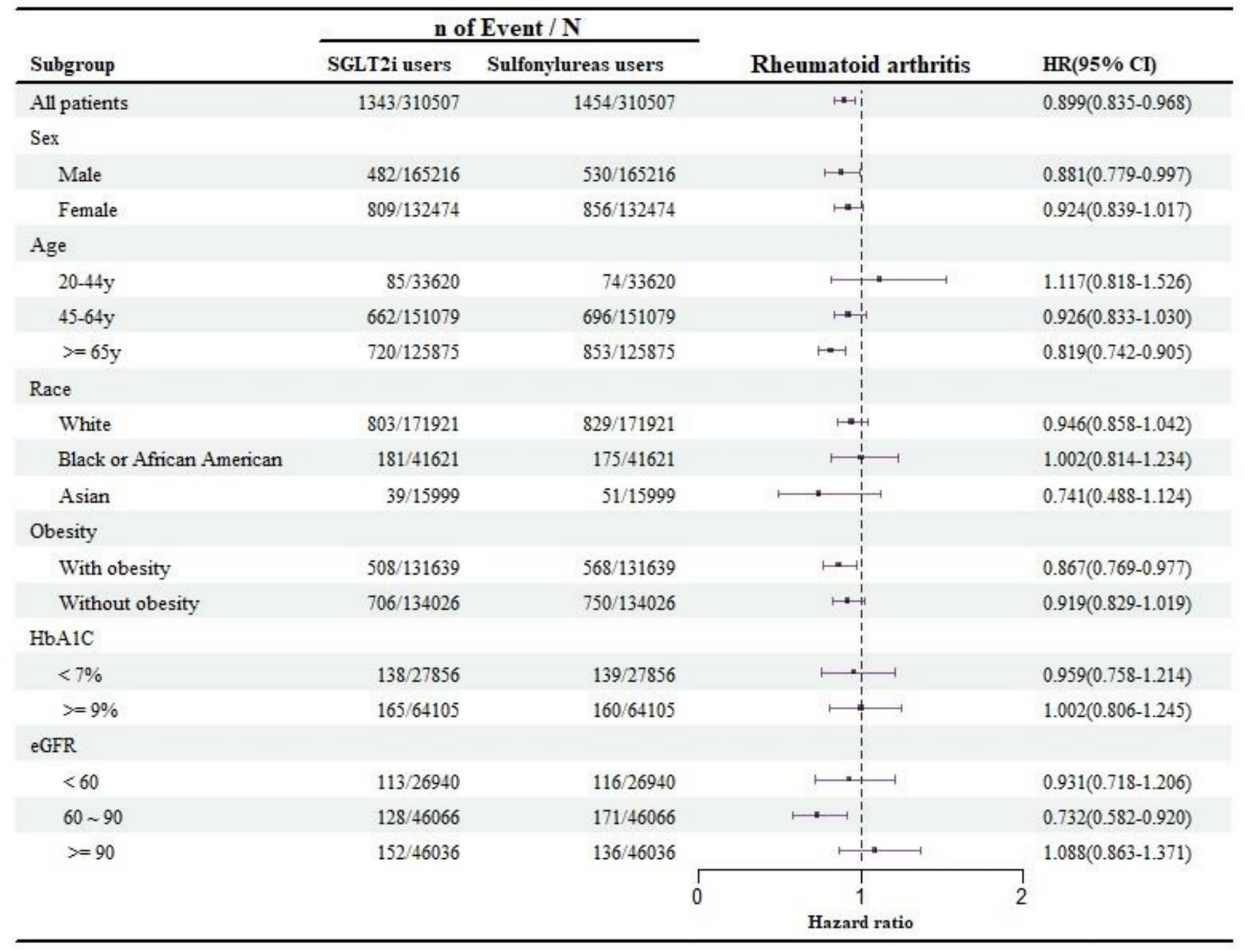
Forest plot of the subgroup analyses. SGLT2i, Sodium-glucose Cotransporter-2 Inhibitors; HbA1c, hemoglobin A1C; eGFR, estimated glomerular filtration rate.

To explore potential variations in RA incidence across different subgroups, we conducted additional analyses within the emulated target trial comparing SGLT2i with sulfonylureas (**Supplementary Tables 8**-**S13**). A summary of these subgroup analyses is shown in **Figure 3**. Our findings suggest that SGLT2i use was associated with a significantly lower RA incidence in certain subgroups, including males, older adults (aged 65 years and above), individuals with obesity, and those with an eGFR of 60–90 mL/min/1.73m² (HR: 0.881, 0.819, 0.867, and 0.732, respectively). However, among individuals with an HbA1c level of less than 7% or greater than or equal to 9% in the year preceding the index date, the RA incidence risk did not differ significantly between SGLT2i and sulfonylurea users (HR: 0.959 for HbA1c < 7%; HR: 1.002 for HbA1c ≥ 9%).

### 3.4 Additional sensitivity analyses: SGLT2i vs. sulfonylureas

The results remained consistent even when propensity score matching was performed using different variable sets (**Supplementary Table 14**). When the study design was modified from an intention-to-treat to a per-protocol approach, SGLT2i use was associated with a slightly lower risk of RA. However, this difference did not reach statistical significance (**Supplementary Table 15**). Importantly, our findings remained robust when the same study design was applied within a research network setting (**Supplementary Table 16**). Additional analyses using different maximum follow-up durations showed that the association between SGLT2i use and a lower risk of incident RA was strongest during the first year (HR, 0.899; 95% CI, 0.835–0.968). The estimated hazard ratios gradually attenuated with longer follow-up durations of 3, 5, and 7 years (HRs, 0.950, 0.972, and 0.976, respectively), and the associations were no longer statistically significant (**Supplementary Table 17**).

## 4 Discussion

These multicenter emulated target trials found that SGLT2i use was significantly associated with a lower risk of RA development compared to sulfonylurea use in patients with T2DM. The subgroup and sensitivity analyses consistently supported these findings. Our findings are consistent with the hypothesis that SGLT2i use may be associated with a lower incidence of RA among patients with T2DM. However, the underlying mechanisms of this association remain unclear. Further research is needed to validate these findings and explore the potential biological pathways involved.

Both hyperglycemia and insulin resistance can potentially increase systemic inflammation and the development of RA (7). Previous research has shown that medications, such as metformin, sulfonylureas, DPP-4i, and TZDs, may reduce the risk of RA development (8–12). Early concerns were raised about the potential association between DPP-4i and arthralgia (22). However, a subsequent meta-analysis demonstrated that DPP-4i did not increase the risk of RA (9). Moreover, several cohort studies and a meta-analysis found that the use of DPP-4i was associated with a significantly lower RA risk (10, 22–25). Additionally, two clinical studies on TZDs suggested that this class of drugs might reduce the RA risk (11, 12). Furthermore, two randomized controlled trials (RCTs) demonstrated that pioglitazone reduced RA disease activity (26, 27). Our study found that the use of SGLT2i was not associated with a significant difference in RA risk compared to the use of DPP-4i and pioglitazone. This finding may be due to the established anti-inflammatory properties and preventive effects of DPP-4i and pioglitazone against RA (10–12).

Our study also revealed that the use of SGLT2i was associated with a significantly lower risk of incident RA compared to sulfonylureas. To date, research on the effects of SGLT2i on autoimmune diseases has been relatively limited. One cohort study reported that SGLT2i use was associated with a significantly lower risk of lupus nephritis compared to DPP-4i in patients with systemic lupus erythematosus and T2DM (19). Additionally, a population-based cohort study indicated that SGLT2i use was not associated with a significant difference in psoriasis risk compared to non-SGLT2i use in patients with T2DM (28). The biological mechanisms underlying the observed association remain incompletely understood. Experimental evidence suggests that SGLT2is possess anti-inflammatory and immunomodulatory properties, which may partially explain the observed association with a lower risk of RA. There has been limited preclinical research on the effects of SGLT2i on joint inflammation. However, some studies suggest that SGLT2i have anti-inflammatory properties and can modulate immune responses. First, SGLT2i may enhance systemic anti-inflammatory effects by inhibiting the activation of the NLRP3 inflammasome and reducing levels of several inflammatory markers, including C-reactive protein, tumor necrosis factor receptor 1, interleukin (IL)-1β, IL-6, IL-17, and IL-23 (13, 15, 29, 30). Second, SGLT2i may enhance fat metabolism and promote the browning of adipose tissue, thereby reducing obesity-induced inflammation by shifting macrophage polarization toward the anti-inflammatory M2 phenotype (13, 14). Third, SGLT2i may activate signaling pathways involving AMP-activated protein kinase (AMPK), sirtuin 1 (SIRT1), sirtuin 3 (SIRT3), and peroxisome proliferator-activated receptor-γ coactivator 1α (PGC-1α), which enhance autophagic flux and help alleviate inflammation (31).

Previous research has shown that SGLT2i may be associated with lower risks of all-cause hospitalization and death (32). Our study also demonstrated that SGLT2i was associated with a lower risk of medical utilization and all-cause mortality compared to sulfonylureas, DPP-4i, and pioglitazone in patients with T2DM.

This study has several notable strengths. First, the application of an emulated target trial design enhances transparency throughout the research process while reducing selection bias and immortal time bias. Second, the use of a new-user, active comparator design minimizes differences due to confounding by indication and other unmeasured confounders. Third, leveraging the extensive TriNetX patient database enabled the identification of a sufficiently large cohort of patients with T2DM treated with SGLT2i, DPP-4i, pioglitazone, and sulfonylureas. The large sample size also facilitated subgroup analyses based on race, obesity, diabetes severity, and kidney disease status. However, the subgroup analyses were exploratory in nature. Because formal interaction testing and adjustment for multiple comparisons were not performed, the subgroup findings should be interpreted cautiously and considered hypothesis-generating rather than confirmatory. Although the active-comparator new-user design may reduce confounding by indication and healthy-user bias compared with conventional observational studies, these biases cannot be completely eliminated.

This study also has several limitations. First, certain potential confounders, such as family history, geographic variations, educational attainment, and inflammatory markers, were not available in the dataset and may have influenced the study outcomes. An additional limitation is the relatively short follow-up period of one year. Because rheumatoid arthritis has a prolonged preclinical phase before clinical diagnosis, some patients with subclinical disease at baseline may have been classified as incident cases during follow-up. Although individuals with a prior diagnosis of RA were excluded and a new-user active-comparator design was adopted to minimize this possibility, reverse causation cannot be completely excluded. Furthermore, differences in healthcare utilization may have contributed to detection bias. To minimize this possibility, healthcare utilization variables were incorporated into the propensity score matching process. In additional analyses using longer follow-up durations, the association between SGLT2i use and RA gradually attenuated over time. Therefore, although the observed association was robust during the first year, it should be interpreted cautiously because reverse causation and delayed diagnosis of pre-existing subclinical RA cannot be completely excluded. Future studies incorporating lag-period analyses and longer follow-up are warranted. Second, rheumatoid arthritis was identified using ICD-10-CM diagnostic codes recorded in electronic health records rather than validated clinical classification criteria or physician chart review. Consequently, some degree of outcome misclassification is possible. However, the same outcome definition was applied consistently across all treatment groups after excluding patients with a prior diagnosis of RA, making any residual misclassification likely to be nondifferential. Such misclassification would be expected to bias the observed associations toward the null rather than generate false-positive findings. Future studies incorporating validated RA case definitions, specialist-confirmed diagnoses, disease-modifying antirheumatic drug prescriptions, or linkage with disease registries are warranted to further validate our findings. Third, we were unable to assess medication adherence, a common limitation of real-world evidence studies, as healthcare providers also lack direct measures of patient adherence. Fourth, an additional limitation relates to the assessment of the proportional hazards assumption. Although formal proportionality tests yielded statistically significant results for some outcomes, these tests are highly sensitive in very large datasets and may detect minor departures from proportionality that are unlikely to be clinically meaningful. Visual inspection of the Kaplan–Meier curves demonstrated generally parallel survival trajectories without substantial crossing, suggesting no major violation of the proportional hazards assumption. Nevertheless, because the TriNetX platform does not support alternative approaches such as time-varying Cox regression or restricted mean survival time analyses, we were unable to further evaluate potential time-varying treatment effects. Future studies using more flexible survival models are warranted to confirm these findings. Finally, despite the use of an emulated target trial framework, a new-user active-comparator design, extensive propensity score matching, and adjustment for a broad range of demographic characteristics, lifestyle factors, diabetes severity, laboratory measurements, comorbidities, concomitant medications, and healthcare utilization, residual confounding cannot be completely excluded. Certain potentially important factors, including smoking intensity, duration of diabetes before cohort entry, healthcare-seeking behaviors beyond routinely recorded healthcare utilization, medication adherence, socioeconomic factors not fully captured in the database, and healthier-user bias, were not directly measurable within the TriNetX platform. Consequently, these unmeasured factors may have influenced the observed associations. Therefore, our findings should be interpreted as associations rather than evidence of causality, and prospective studies and randomized clinical trials are warranted to further validate these findings.

This multicenter emulated target trial found that SGLT2i use was associated with a lower risk of incident rheumatoid arthritis compared with sulfonylurea use during the one-year follow-up among patients with T2DM. However, the observed association attenuated with longer follow-up and should therefore be interpreted cautiously. Given the observational study design, these findings represent associations rather than evidence of causality. Further studies with longer follow-up durations and lag-period analyses are warranted to confirm these findings and clarify the underlying mechanisms.

## Data Availability

The data that support the findings of this study are not publicly available due to privacy reasons, but are available from accessing the website TriNetX upon reasonable request: https://trinetx.com/?mc_cid=7e2ecd5bc5&mc_eid=%5BUNIQID%5D.
