# Supplementary material for: The association between SGLT2 inhibitors and rheumatoid arthritis risk in type 2 diabetes: findings from large-scale emulated target trials
Source: Front Immunol. 2026 Aug 4;17:1867117. doi: 10.3389/fimmu.2026.1867117 (PMC13481369; doi:10.3389/fimmu.2026.1867117)
Supplement: Supplementary file 1 [file Table1.docx]

Supplementary Material

# 1 Supplementary Tables

**Table S1.** Key features of the emulated randomized clinical trial and the emulated target trials evaluating rheumatoid arthritis risk in SGLT2i and non-SGLT2i users with T2DM

|  | **CAROLINA** | **Emulated target trial 1** | **Emulated target trial 2** | **Emulated target trial 3** |
| --- | --- | --- | --- | --- |
| Arms | 1. Linagliptin 2. Glimepiride | 1. SGLT2i  2. Sulfonylureas | 1. SGLT2i  2. DPP-4i | 1. SGLT2i  2. Pioglitazone |
| Primary outcomes | Cardiovascular  death, non-fatal stroke, or non-fatal myocardial infarction. | The incidence of rheumatoid arthritis | The incidence of rheumatoid arthritis | The incidence of rheumatoid arthritis |
| Inclusion criteria | HbA1c of 6.5% to 8.5% and high cardiovascular risk (atherosclerotic cardiovascular disease, multiple cardiovascular risk factors, age 70 years or older, and evidence of microvascular complications). | Individuals with T2DM who have no previous diagnosis of rheumatoid arthritis. | Individuals with T2DM who have no previous diagnosis of rheumatoid arthritis. | Individuals with T2DM who have no previous diagnosis of rheumatoid arthritis. |
| Exclusion criteria | Type 1 diabetes mellitus, congestive heart failure classified as NYHA class III or IV, and acute or chronic metabolic acidosis. Ongoing treatment with systemic corticosteroids at the time of informed consent or a planned initiation of such therapy. | Individuals under 20 years of age, those with a prior diagnosis of type 1 diabetes mellitus, or those who have undergone dialysis or kidney transplantation. | Individuals under 20 years of age, those with a prior diagnosis of type 1 diabetes mellitus, or those who have undergone dialysis or kidney transplantation. | Individuals under 20 years of age, those with a prior diagnosis of type 1 diabetes mellitus, or those who have undergone dialysis or kidney transplantation. |
| Sample size | 6042 | 621,014 | 412,138 | 161,692 |
| Location | 607 sites in 43 countries. | US Collaborative Network | US Collaborative Network | US Collaborative Network |
| Year final results reported | 2019 | 2025 | 2025 | 2025 |

SGLT2i: Sodium-glucose Cotransporter-2 Inhibitors; DPP-4 = dipeptidyl peptidase-4; T2DM = type 2 diabetes mellitus; CAROLINA = Cardiovascular Outcome Study of Linagliptin vs Glimepiride in Type 2 Diabetes; HbA1c = glycated hemoglobin; NYHA = New York Heart Association.

**Table S2.** Specification and emulation of three target trials on the use of SGLT2i in T2DM, with the risk of rheumatoid arthritis, using the TriNetX collaborative network

| **Protocol** | **Specification of Target Trials** | **Emulation of Target Trials** |
| --- | --- | --- |
| **Eligibility criteria** | - Age ≧20 years between 1 January 2016 and 30 June 2024. - Diagnosed with type 2 diabetes mellitus - Not diagnosed with type 1 diabetes mellitus. - Not on dialysis or kidney transplantation prior to enrolment in the study. | Same as for the target trials. |
| **Treatment strategies** | For the target trial comparing SGLT2i vs sulfonylureas:   1) Initial use of SGLT2i at baseline (index date) and no sulfonylureas initiated on the same day. 2) Initial use of sulfonylureas at baseline (index date) and no SGLT2i initiated on the same day.   For the target trial comparing SGLT2i vs DPP-4i:  1) Initial use of SGLT2i at baseline (index date) and no DPP-4i initiated on the same day.  2) Initial use of DPP-4i at baseline (index date) and no SGLT2i initiated on the same day.  For the target trial comparing SGLT2i vs pioglitazone:  1) Initial use of SGLT2i at baseline (index date) and no pioglitazone initiated on the same day.  2) Initial use of pioglitazone at baseline (index date) and no SGLT2i initiated on the same day. | As per target trials, we defined the date of medication initiation as the date of the first filled prescription. |
| **Treatment assignment** | Individuals were randomly assigned to a treatment strategy at baseline. | Individuals were assigned to the treatment strategy corresponding to their first filled prescription, assuming randomization based on baseline covariates. |
| **Outcomes** | The incidence of rheumatoid arthritis, medical utilization, and all-cause mortality. | Same as for the target trials. |
| **Follow-up** | Follow-up of each individual began at the time of treatment assignment and continued until the occurrence of outcomes, death, or the end of the study on 30 June 2024, whichever occurred first. | Same as for the target trials. |
| **Causal contrast of interest** | Intention-to-treat. | Observational analog to intention-to-treat effect. |
| **Statistical analysis** | Kaplan-Meier methods were used to determine the cumulative incidence for each treatment strategy during follow-up. The log-rank test was used to compare the cumulative incidence between treatment strategies.  Cox regression models were used to compare the risk of outcomes between treatment strategies. | Same as for the target trial except that observational analogs of intention-to-treat analyses require additional adjustment for confounding. |

SGLT2i: Sodium-glucose Cotransporter-2 Inhibitors; DPP-4 = dipeptidyl peptidase-4; T2DM = type 2 diabetes mellitus.

**Table S3.** Diseases and associated ICD-10-CM and CPT codes

| **Disease/ condition** | **ICD-10-CM codes** | **CPT codes** |
| --- | --- | --- |
| Rheumatoid arthritis | M05, M06 |  |
| Type 2 diabetes mellitus | E08–E13, except E10 |  |
| Type 1 diabetes | E10 |  |
|  |  |  |
|  |  |  |
| Dialysis | Z49.3, Z99.2 | 90935, 90937, 90945, 90947, 90989, 90999, 90960, 90961, 90962, 90965, 90966, 90957, 90958, 90959, 90969, 90970, 1012740 |
| kidney transplant | Z94.0 |  |
| All-cause hospitalization |  | 1013659, 1013729 |
| Office or Other Outpatient Services |  | 1013626 |
| Hospital Inpatient Services |  | 1013659 |
| Emergency Department Services |  | 1013711 |
| Critical Care Services |  | 1013729 |
| Hispanic or Latino |  | 85865 |
| Not Hispanic or Latino |  | 104582 |
| American Indian or Alaska Native |  | 1002-5 |
| Asian |  | 46997 |
| African American |  | 56370 |
| Native Hawaiian or Other Pacific Islander |  | 64498 |
| White |  | 75301 |
| Other Race |  | 84373 |
| Socioeconomic and psychosocial circumstances | Z55-Z65 |  |
| Problems related to education and literacy | Z55 |  |
| Problems related to employment and unemployment | Z56 |  |
| Occupational exposure to risk factors | Z57 |  |
| Problems related to housing and economic circumstances | Z59 |  |
| Tobacco use | Z72.0 |  |
| Nicotine dependence | F17 |  |
| Type 2 diabetes mellitus with hyperosmolarity | E11.0 |  |
| Type 2 diabetes mellitus with ketoacidosis | E11.1 |  |
| Type 2 diabetes mellitus with kidney complications | E11.2 |  |
| Type 2 diabetes mellitus with ophthalmic complications | E11.5 |  |
| Type 2 diabetes mellitus with neurological complications | E11.4 |  |
| Type 2 diabetes mellitus with circulatory complications | E11.5 |  |
| Type 2 diabetes mellitus with other specified complications | E11.6 |  |
| Type 2 diabetes mellitus with unspecified complications | E11.8 |  |
| Type 2 diabetes mellitus without complications | E11.9 |  |
| Overweight and obesity | E66 |  |
| Alcohol related disorders | F10 |  |
| Hypertensive diseases | I10-I1A |  |
| Hyperlipidemia | E78 |  |
| Ischemic heart diseases | I20-I25 |  |
| Cerebrovascular diseases | I60-I69 |  |
| Heart failure | I50 |  |
| Heart failure exacerbation | Z79.899, J81 |  |
| Diseases of arteries, arterioles, and capillaries | I70-I79 |  |
| Chronic lower respiratory diseases | J44-J4A |  |
| Asthma | J45 |  |
| Diseases of liver | K70-K77 |  |
| Viral hepatitis | B15-B19 |  |
| Neoplasms | C00-D49 |  |
| Chronic kidney disease | N18 |  |
| Unspecified kidney failure | N19 |  |
| Proteinuria, unspecified | R80.9 |  |
| Depressive episode | F32 |  |
| Major depressive disorder, recurrent | F33 |  |
| Psoriasis | L40 |  |
| Systemic lupus erythematosus | M32 |  |
| Ankylosing spondylitis | M45 |  |

**Table S4.** Baseline characteristics of study subjects: SGLT2i vs. DPP-4i

| Variables | Before PSM | | | After PSM | | |
| --- | --- | --- | --- | --- | --- | --- |
|  | SGLT2i users  (n=331037) | DPP-4i users  (n=217044) | SMD | SGLT2i users  (n=206069) | DPP-4i users  (n=206069) | SMD |
| Age at Index, y |  |  |  |  |  |  |
| Mean ± SD | 60.6 ± 12.7 | 62.0 ± 13.3 | **0.107** | 61.4 ± 12.5 | 61.5 ± 13.3 | 0.003 |
| Sex, n (%) |  |  |  |  |  |  |
| Male | 182462 (55.1) | 106369 (49.0) | **0.123** | 103771 (50.4) | 104065 (50.5) | 0.003 |
| Female | 135437 (40.9) | 103730 (47.8) | **0.139** | 95522 (46.4) | 95154 (46.2) | 0.004 |
| Unknown Gender | 13138 (4.0) | 6945 (3.2) | 0.041 | 6776 (3.3) | 6850 (3.3) | 0.002 |
| Race, n (%) |  |  |  |  |  |  |
| White | 198825 (60.1) | 126361 (58.2) | 0.037 | 120894 (58.7) | 120941 (58.7) | 0.000 |
| Black or African American | 53448 (16.1) | 35741 (16.5) | 0.009 | 33908 (16.5) | 33854 (16.4) | 0.001 |
| Other Race | 21290 (6.4) | 15066 (6.9) | 0.020 | 14128 (6.9) | 14240 (6.9) | 0.002 |
| Asian | 18632 (5.6) | 14512 (6.7) | 0.044 | 13237 (6.4) | 13127 (6.4) | 0.002 |
| Native Hawaiian or Other Pacific Islander | 2318 (0.7) | 1255 (0.6) | 0.015 | 1209 (0.6) | 1202 (0.6) | 0.000 |
| American Indian or Alaska Native | 1323 (0.4) | 855 (0.4) | 0.001 | 790 (0.4) | 832 (0.4) | 0.003 |
| Unknown Race | 35201 (10.6) | 23254 (10.7) | 0.003 | 21903 (10.6) | 21873 (10.6) | 0.000 |
| Social economic status, n (%) |  |  |  |  |  |  |
| Persons with potential health hazards related to socioeconomic and psychosocial circumstances | 6692 (2.0) | 3398 (1.6) | 0.034 | 3218 (1.6) | 3261 (1.6) | 0.002 |
| Lifestyles, n (%) |  |  |  |  |  |  |
| Nicotine dependence | 31622 (9.6) | 18539 (8.5) | 0.035 | 17433 (8.5) | 17609 (8.5) | 0.003 |
| Personal history of nicotine dependence | 33010 (10.0) | 17421 (8.0) | 0.068 | 16502 (8.0) | 16720 (8.1) | 0.004 |
| Tobacco use | 10729 (3.2) | 5531 (2.5) | 0.041 | 5314 (2.6) | 5367 (2.6) | 0.002 |
| Alcohol related disorders | 6373 (1.9) | 3570 (1.6) | 0.021 | 3252 (1.6) | 3393 (1.6) | 0.005 |
| DM severity, n (%) |  |  |  |  |  |  |
| Type 2 diabetes mellitus without complications | 208027 (62.8) | 133600 (61.6) | 0.027 | 127271 (61.8) | 126546 (61.4) | 0.007 |
| Type 2 diabetes mellitus with other specified complications | 120621 (36.4) | 67631 (31.2) | **0.112** | 65746 (31.9) | 65518 (31.8) | 0.002 |
| Type 2 diabetes mellitus with kidney complications | 45727 (13.8) | 24757 (11.4) | 0.073 | 23496 (11.4) | 23362 (11.3) | 0.002 |
| Type 2 diabetes mellitus with neurological complications | 38049 (11.5) | 21274 (9.8) | 0.055 | 20593 (10.0) | 20535 (10.0) | 0.001 |
| Type 2 diabetes mellitus with circulatory complications | 22226 (6.7) | 10970 (5.1) | 0.071 | 10821 (5.3) | 10670 (5.2) | 0.003 |
| Type 2 diabetes mellitus with unspecified complications | 13726 (4.1) | 9091 (4.2) | 0.002 | 8658 (4.2) | 8548 (4.1) | 0.003 |
| Type 2 diabetes mellitus with ophthalmic complications | 13762 (4.2) | 7037 (3.2) | 0.048 | 6970 (3.4) | 6913 (3.4) | 0.002 |
| Type 2 diabetes mellitus with hyperosmolarity | 3580 (1.1) | 2190 (1.0) | 0.007 | 1995 (1.0) | 2052 (1.0) | 0.003 |
| Type 2 diabetes mellitus with ketoacidosis | 2359 (0.7) | 1642 (0.8) | 0.005 | 1493 (0.7) | 1498 (0.7) | 0.000 |
| Medical utilization, n (%) |  |  |  |  |  |  |
| Office or other outpatient services | 169112 (51.1) | 93552 (43.1) | **0.160** | 92538 (44.9) | 91994 (44.6) | 0.005 |
| Emergency department services | 62101 (18.8) | 41065 (18.9) | 0.004 | 36903 (17.9) | 37248 (18.1) | 0.004 |
| Hospital inpatient and observation care services | 48508 (14.7) | 28346 (13.1) | 0.046 | 25454 (12.4) | 25916 (12.6) | 0.007 |
| Preventive medicine services | 29123 (8.8) | 13905 (6.4) | 0.090 | 14022 (6.8) | 13848 (6.7) | 0.003 |
| Comorbidities, n (%) |  |  |  |  |  |  |
| Hypertensive diseases | 218803 (66.1) | 133888 (61.7) | 0.092 | 127083 (61.7) | 127056 (61.7) | 0.000 |
| Disorders of lipoprotein metabolism and other lipidemias | 200647 (60.6) | 115363 (53.2) | **0.151** | 112038 (54.4) | 111597 (54.2) | 0.004 |
| Overweight and obesity | 92406 (27.9) | 45278 (20.9) | **0.165** | 44631 (21.7) | 44572 (21.6) | 0.001 |
| Ischemic heart diseases | 79051 (23.9) | 37762 (17.4) | **0.161** | 36546 (17.7) | 36880 (17.9) | 0.004 |
| Chronic lower respiratory diseases | 47922 (14.5) | 28745 (13.2) | 0.036 | 26836 (13.0) | 27016 (13.1) | 0.003 |
| Neoplasms | 43620 (13.2) | 27760 (12.8) | 0.012 | 25955 (12.6) | 26254 (12.7) | 0.004 |
| Chronic kidney disease | 47999 (14.5) | 27104 (12.5) | 0.059 | 25406 (12.3) | 25464 (12.4) | 0.001 |
| Depressive episode | 32292 (9.8) | 20212 (9.3) | 0.015 | 18861 (9.2) | 18985 (9.2) | 0.002 |
| Heart failure | 57389 (17.3) | 18579 (8.6) | **0.264** | 18553 (9.0) | 18558 (9.0) | 0.000 |
| Diseases of arteries, arterioles and capillaries | 29781 (9.0) | 16001 (7.4) | 0.059 | 15214 (7.4) | 15259 (7.4) | 0.001 |
| Acute kidney failure | 25070 (7.6) | 17360 (8.0) | 0.016 | 14771 (7.2) | 14694 (7.1) | 0.001 |
| Cerebrovascular diseases | 23182 (7.0) | 16068 (7.4) | 0.015 | 14480 (7.0) | 14371 (7.0) | 0.002 |
| Diseases of liver | 24115 (7.3) | 13162 (6.1) | 0.049 | 12660 (6.1) | 12700 (6.2) | 0.001 |
| Proteinuria, unspecified | 15824 (4.8) | 5717 (2.6) | **0.114** | 5913 (2.9) | 5705 (2.8) | 0.006 |
| Major depressive disorder, recurrent | 9827 (3.0) | 5069 (2.3) | 0.039 | 4914 (2.4) | 4973 (2.4) | 0.002 |
| Viral hepatitis | 3101 (0.9) | 2326 (1.1) | 0.014 | 2088 (1.0) | 2061 (1.0) | 0.001 |
| Psoriasis | 3459 (1.0) | 1738 (0.8) | 0.026 | 1694 (0.8) | 1713 (0.8) | 0.001 |
| Systemic lupus erythematosus | 741 (0.2) | 443 (0.2) | 0.004 | 439 (0.2) | 418 (0.2) | 0.002 |
| Unspecified kidney failure | 523 (0.2) | 572 (0.3) | 0.023 | 411 (0.2) | 387 (0.2) | 0.003 |
| Ankylosing spondylitis | 302 (0.1) | 157 (0.1) | 0.007 | 158 (0.1) | 153 (0.1) | 0.001 |
| Comedication, n (%) |  |  |  |  |  |  |
| Metformin | 180095 (54.4) | 128089 (59.0) | 0.093 | 112770 (54.7) | 122744 (59.6) | 0.098 |
| Insulins and analogues | 119816 (36.2) | 73709 (34.0) | 0.047 | 70571 (34.2) | 69066 (33.5) | 0.015 |
| Sulfonylureas | 65898 (19.9) | 57351 (26.4) | **0.155** | 42656 (20.7) | 54594 (26.5) | **0.137** |
| Glucagon-like peptide-1 analogues | 66739 (20.2) | 15101 (7.0) | **0.393** | 39807 (19.3) | 14709 (7.1) | **0.365** |
| Thiazolidinediones | 13264 (4.0) | 9645 (4.4) | 0.022 | 8809 (4.3) | 9203 (4.5) | 0.009 |
| HMG CoA reductase inhibitors | 203007 (61.3) | 127834 (58.9) | 0.050 | 122929 (59.7) | 121359 (58.9) | 0.016 |
| Beta blocking agents | 123904 (37.4) | 74653 (34.4) | 0.063 | 69352 (33.7) | 70310 (34.1) | 0.010 |
| Diuretics | 124720 (37.7) | 73692 (34.0) | 0.078 | 69168 (33.6) | 69910 (33.9) | 0.008 |
| ACE inhibitors | 99436 (30.0) | 68095 (31.4) | 0.029 | 60283 (29.3) | 64941 (31.5) | 0.049 |
| Angiotensin II receptor blockers | 94701 (28.6) | 50262 (23.2) | **0.125** | 54677 (26.5) | 47395 (23.0) | 0.082 |
| Corticosteroids for systemic use | 90801 (27.4) | 55882 (25.7) | 0.038 | 53036 (25.7) | 53064 (25.8) | 0.000 |
| Proton pump inhibitors | 84762 (25.6) | 58230 (26.8) | 0.028 | 51442 (25.0) | 54689 (26.5) | 0.036 |
| Calcium channel blockers | 84184 (25.4) | 57265 (26.4) | 0.022 | 50736 (24.6) | 53488 (26.0) | 0.031 |
| Aspirin | 85631 (25.9) | 57104 (26.3) | 0.010 | 48886 (23.7) | 53533 (26.0) | 0.052 |
| NSAIDs | 75218 (22.7) | 50560 (23.3) | 0.014 | 46474 (22.6) | 48119 (23.4) | 0.019 |
| Alpha-adrenoreceptor antagonists | 3809 (1.2) | 2641 (1.2) | 0.006 | 2256 (1.1) | 2503 (1.2) | 0.011 |
| Laboratory, n (%) |  |  |  |  |  |  |
| Hemoglobin A1c/Hemoglobin. total in Blood (%) |  |  |  |  |  |  |
| < 7 | 75411 (22.8) | 38400 (17.7) | **0.127** | 36939 (17.9) | 37607 (18.3) | 0.008 |
| ≧9 | 69926 (21.1) | 36678 (16.9) | **0.108** | 36654 (17.8) | 36135 (17.5) | 0.007 |
| estimated Glomerular filtration rate (eGFR^b^, ml/min/1.73m^2^) |  |  |  |  |  |  |
| < 60 | 83960 (25.4) | 51068 (23.5) | 0.043 | 47140 (22.9) | 47560 (23.1) | 0.005 |
| 60 ~ 90 | 131841 (39.8) | 72040 (33.2) | **0.138** | 69683 (33.8) | 70191 (34.1) | 0.005 |
| Body mass index (BMI, kg/m^2^) |  |  |  |  |  |  |
| ≧30 | 157152 (47.5) | 82866 (38.2) | **0.189** | 81662 (39.6) | 81716 (39.7) | 0.001 |
| Rheumatoid factor [units/volume] in serum or plasma (IU/mL) |  |  |  |  |  |  |
| ≧20 | 170 (0.1) | 107 (0.0) | 0.001 | 82 (0.0) | 101 (0.0) | 0.004 |

Note:

Bold font represents a standardized difference was more than 0.1.

If the patient is less or equal to 10, results show the count as 10.

PSM: Propensity score matching, SGLT2i: Sodium-glucose Cotransporter-2 Inhibitors, DPP-4i: Dipeptidyl peptidase 4 inhibitors, SMD: Standardized mean difference, SD: Standard deviation, DM: Diabetes mellitus, ACE: Angiotensin-converting enzyme, HMG CoA: β-Hydroxy β-methylglutaryl-CoA, NSAIDs: Anti-inflammatory and anti-rheumatic products, non-steroids.

a.	Propensity score matching was performed on age at index, sex, race, socioeconomic status, DM severity, lifestyles, medical utilization, comorbidities, and HbA1C, eGFR, BMI, rheumatoid factor.

b.	Predicted by Creatinine-based formula (modification of diet in renal disease, MDRD).

**Table S5**. Baseline characteristics of study subjects: SGLT2i vs. Pioglitazone

| Variables | Before PSM | | | After PSM | | |
| --- | --- | --- | --- | --- | --- | --- |
|  | SGLT2i  users  (n=378850) | Pioglitazone users  (n=815930) | SMD | SGLT2i  users  (n=80846) | Pioglitazone users  (n=80846) | SMD |
| Age at Index, y |  |  |  |  |  |  |
| Mean ± SD | 60.6 ± 12.4 | 62.2 ± 12.3 | **0.127** | 61.7 ± 11.7 | 62.1 ± 12.3 | 0.029 |
| Sex, n (%) |  |  |  |  |  |  |
| Male | 209336 (55.3) | 44838 (55.0) | 0.006 | 44567 (55.1) | 44433 (55.0) | 0.003 |
| Female | 154895 (40.9) | 35005 (42.9) | 0.041 | 34556 (42.7) | 34663 (42.9) | 0.003 |
| Unknown Gender | 14619 (3.9) | 1750 (2.1) | **0.101** | 1723 (2.1) | 1750 (2.2) | 0.002 |
| Race, n (%) |  |  |  |  |  |  |
| White | 223453 (59.0) | 51453 (63.1) | 0.084 | 51149 (63.3) | 50904 (63.0) | 0.006 |
| Black or African American | 63840 (16.9) | 10824 (13.3) | 0.100 | 10389 (12.9) | 10791 (13.3) | 0.015 |
| Asian | 22941 (6.1) | 5316 (6.5) | 0.019 | 5450 (6.7) | 5252 (6.5) | 0.010 |
| Other Race | 22041 (5.8) | 5203 (6.4) | 0.023 | 5141 (6.4) | 5152 (6.4) | 0.001 |
| Native Hawaiian or Other Pacific Islander | 2369 (0.6) | 293 (0.4) | 0.038 | 331 (0.4) | 292 (0.4) | 0.008 |
| American Indian or Alaska Native | 1439 (0.4) | 316 (0.4) | 0.001 | 307 (0.4) | 315 (0.4) | 0.002 |
| Unknown Race | 42767 (11.3) | 8188 (10.0) | 0.041 | 8079 (10.0) | 8140 (10.1) | 0.003 |
| Social economic status, n (%) |  |  |  |  |  |  |
| Persons with potential health hazards related to socioeconomic and psychosocial circumstances | 7479 (2.0) | 1023 (1.3) | 0.057 | 1012 (1.3) | 1019 (1.3) | 0.001 |
| Lifestyles, n (%) |  |  |  |  |  |  |
| Personal history of nicotine dependence | 37597 (9.9) | 5975 (7.3) | 0.093 | 5763 (7.1) | 5913 (7.3) | 0.007 |
| Nicotine dependence | 33743 (8.9) | 5248 (6.4) | 0.093 | 5048 (6.2) | 5225 (6.5) | 0.009 |
| Tobacco use | 11979 (3.2) | 1647 (2.0) | 0.072 | 1604 (2.0) | 1646 (2.0) | 0.004 |
| Alcohol related disorders | 7107 (1.9) | 881 (1.1) | 0.066 | 815 (1.0) | 879 (1.1) | 0.008 |
| DM severity, n (%) |  |  |  |  |  |  |
| Type 2 diabetes mellitus without complications | 233549 (61.6) | 47712 (58.5) | 0.065 | 46873 (58.0) | 47268 (58.5) | 0.010 |
| Type 2 diabetes mellitus with other specified complications | 136379 (36.0) | 26760 (32.8) | 0.067 | 26632 (32.9) | 26629 (32.9) | 0.000 |
| Type 2 diabetes mellitus with neurological complications | 41845 (11.0) | 8107 (9.9) | 0.036 | 7867 (9.7) | 8056 (10.0) | 0.008 |
| Type 2 diabetes mellitus with kidney complications | 51023 (13.5) | 8107 (9.9) | **0.110** | 7587 (9.4) | 8053 (10.0) | 0.020 |
| Type 2 diabetes mellitus with circulatory complications | 24304 (6.4) | 3730 (4.6) | 0.081 | 3619 (4.5) | 3719 (4.6) | 0.006 |
| Type 2 diabetes mellitus with unspecified complications | 15863 (4.2) | 3173 (3.9) | 0.015 | 3017 (3.7) | 3168 (3.9) | 0.010 |
| Type 2 diabetes mellitus with ophthalmic complications | 15188 (4.0) | 2836 (3.5) | 0.028 | 2816 (3.5) | 2829 (3.5) | 0.001 |
| Type 2 diabetes mellitus with hyperosmolarity | 4083 (1.1) | 722 (0.9) | 0.020 | 711 (0.9) | 719 (0.9) | 0.001 |
| Type 2 diabetes mellitus with ketoacidosis | 2834 (0.7) | 588 (0.7) | 0.003 | 579 (0.7) | 583 (0.7) | 0.001 |
| Medical utilization, n (%) |  |  |  |  |  |  |
| Office or other outpatient services | 193600 (51.1) | 36614 (44.9) | **0.125** | 36453 (45.1) | 36482 (45.1) | 0.001 |
| Emergency department services | 73513 (19.4) | 14237 (17.4) | 0.050 | 13512 (16.7) | 13960 (17.3) | 0.015 |
| Hospital inpatient and observation care services | 52745 (13.9) | 6905 (8.5) | **0.174** | 6430 (8.0) | 6843 (8.5) | 0.019 |
| Preventive medicine services | 31644 (8.4) | 4621 (5.7) | **0.105** | 4680 (5.8) | 4620 (5.7) | 0.003 |
| Comorbidities, n (%) |  |  |  |  |  |  |
| Hypertensive diseases | 247104 (65.2) | 48478 (59.4) | **0.120** | 47220 (58.4) | 48063 (59.5) | 0.021 |
| Disorders of lipoprotein metabolism and other lipidemias | 226038 (59.7) | 44755 (54.9) | 0.097 | 44083 (54.5) | 44415 (54.9) | 0.008 |
| Overweight and obesity | 101673 (26.8) | 17242 (21.1) | **0.134** | 16856 (20.9) | 17178 (21.2) | 0.010 |
| Ischemic heart diseases | 89443 (23.6) | 10546 (12.9) | **0.279** | 10164 (12.6) | 10534 (13.0) | 0.014 |
| Neoplasms | 50077 (13.2) | 9407 (11.5) | 0.051 | 8946 (11.1) | 9358 (11.6) | 0.016 |
| Chronic lower respiratory diseases | 52332 (13.8) | 7942 (9.7) | **0.127** | 7570 (9.4) | 7904 (9.8) | 0.014 |
| Chronic kidney disease | 53828 (14.2) | 8066 (9.9) | **0.133** | 7328 (9.1) | 8007 (9.9) | 0.029 |
| Depressive episode | 35735 (9.4) | 6478 (7.9) | 0.053 | 6276 (7.8) | 6435 (8.0) | 0.007 |
| Diseases of liver | 27907 (7.4) | 4730 (5.8) | 0.063 | 4635 (5.7) | 4716 (5.8) | 0.004 |
| Cerebrovascular diseases | 27206 (7.2) | 4701 (5.8) | 0.058 | 4458 (5.5) | 4631 (5.7) | 0.009 |
| Diseases of arteries, arterioles and capillaries | 33757 (8.9) | 4498 (5.5) | **0.132** | 4218 (5.2) | 4484 (5.5) | 0.015 |
| Heart failure | 64994 (17.2) | 3383 (4.1) | **0.431** | 2869 (3.5) | 3383 (4.2) | 0.033 |
| Proteinuria, unspecified | 17281 (4.6) | 2383 (2.9) | 0.087 | 2406 (3.0) | 2380 (2.9) | 0.002 |
| Major depressive disorder, recurrent | 10550 (2.8) | 1751 (2.1) | 0.041 | 1649 (2.0) | 1748 (2.2) | 0.009 |
| Psoriasis | 3782 (1.0) | 606 (0.7) | 0.028 | 593 (0.7) | 605 (0.7) | 0.002 |
| Viral hepatitis | 3638 (1.0) | 528 (0.6) | 0.035 | 516 (0.6) | 527 (0.7) | 0.002 |
| Systemic lupus erythematosus | 879 (0.2) | 117 (0.1) | 0.020 | 127 (0.2) | 117 (0.1) | 0.003 |
| Unspecified kidney failure | 554 (0.1) | 130 (0.2) | 0.003 | 81 (0.1) | 122 (0.2) | 0.014 |
| Ankylosing spondylitis | 331 (0.1) | 54 (0.1) | 0.008 | 64 (0.1) | 54 (0.1) | 0.005 |
| Comedication, n (%) |  |  |  |  |  |  |
| Metformin | 210784 (55.6) | 48358 (59.3) | 0.073 | 48419 (59.9) | 47944 (59.3) | 0.012 |
| Dipeptidyl peptidase 4 inhibitors | 52158 (13.8) | 15460 (18.9) | **0.140** | 14299 (17.7) | 15312 (18.9) | 0.032 |
| Glucagon-like peptide-1 analogues | 77781 (20.5) | 14810 (18.2) | 0.060 | 16932 (20.9) | 14775 (18.3) | 0.067 |
| Insulins and analogues | 138801 (36.6) | 26509 (32.5) | 0.087 | 26799 (33.1) | 26263 (32.5) | 0.014 |
| ACE inhibitors | 114323 (30.2) | 28162 (34.5) | 0.093 | 25119 (31.1) | 27871 (34.5) | 0.073 |
| Beta blocking agents | 145181 (38.3) | 24868 (30.5) | **0.166** | 25059 (31.0) | 24584 (30.4) | 0.013 |
| Diuretics | 144602 (38.2) | 25216 (30.9) | **0.153** | 24790 (30.7) | 24939 (30.8) | 0.004 |
| Angiotensin II receptor blockers | 113550 (30.0) | 19738 (24.2) | **0.130** | 21129 (26.1) | 19536 (24.2) | 0.045 |
| Corticosteroids for systemic use | 104964 (27.7) | 20487 (25.1) | 0.059 | 19683 (24.3) | 20345 (25.2) | 0.019 |
| Sulfonylureas | 74746 (19.7) | 28696 (35.2) | **0.351** | 19310 (23.9) | 28415 (35.1) | **0.249** |
| Proton pump inhibitors | 98468 (26.0) | 20561 (25.2) | 0.018 | 19303 (23.9) | 20351 (25.2) | 0.030 |
| Calcium channel blockers | 97878 (25.8) | 19227 (23.6) | 0.053 | 18560 (23.0) | 19030 (23.5) | 0.014 |
| HMG CoA reductase inhibitors | 236762 (62.5) | 51994 (63.7) | 0.025 | 49180 (60.8) | 51500 (63.7) | 0.059 |
| NSAIDs | 86107 (22.7) | 19451 (23.8) | 0.026 | 18181 (22.5) | 19312 (23.9) | 0.033 |
| Aspirin | 98774 (26.1) | 18448 (22.6) | 0.081 | 17579 (21.7) | 18195 (22.5) | 0.018 |
| Alpha-adrenoreceptor antagonists | 4329 (1.1) | 983 (1.2) | 0.006 | 859 (1.1) | 965 (1.2) | 0.012 |
| Laboratory, n (%) |  |  |  |  |  |  |
| Hemoglobin A1c/Hemoglobin. total in Blood (%) |  |  |  |  |  |  |
| < 7 | 84510 (22.3) | 13655 (16.7) | **0.141** | 12811 (15.8) | 13598 (16.8) | 0.026 |
| ≧9 | 78538 (20.7) | 15641 (19.2) | 0.039 | 15623 (19.3) | 15589 (19.3) | 0.001 |
| estimated Glomerular filtration rate (eGFR^b^, ml/min/1.73m^2^) |  |  |  |  |  |  |
| < 60 | 92422 (24.4) | 16115 (19.8) | **0.112** | 14890 (18.4) | 15966 (19.7) | 0.034 |
| 60 ~ 90 | 148303 (39.1) | 27117 (33.2) | **0.123** | 26243 (32.5) | 26999 (33.4) | 0.020 |
| ≧90 | 110053 (29.0) | 20041 (24.6) | **0.101** | 19868 (24.6) | 19979 (24.7) | 0.003 |
| Body mass index (BMI, kg/m^2^) |  |  |  |  |  |  |
| ≧30 | 177696 (46.9) | 33719 (41.3) | **0.113** | 33036 (40.9) | 33534 (41.5) | 0.013 |
| Rheumatoid factor [Units/volume] in Serum or Plasma (IU/mL) |  |  |  |  |  |  |
| ≧30 | 114 (0.0) | 12 (0.0) | 0.010 | 10 (0.0) | 12 (0.0) | 0.002 |

Note:

Bold font represents a standardized difference was more than 0.1.

If the patient is less or equal to 10, results show the count as 10.

PSM: Propensity score matching, SGLT2i: Sodium-glucose Cotransporter-2 Inhibitors, SMD: Standardized mean difference, SD: Standard deviation, DM: Diabetes mellitus, ACE: Angiotensin-converting enzyme, HMG CoA: β-Hydroxy β-methylglutaryl-CoA, NSAIDs: Anti-inflammatory and anti-rheumatic products, non-steroids.

a.	Propensity score matching was performed on age at index, current age, sex, race, socioeconomic status, DM severity, lifestyles, medical utilization, comorbidities, and HbA1C, eGFR, BMI, rheumatoid factor.

b.	Predicted by Creatinine-based formula (modification of diet in renal disease, MDRD).

**Table S6.** Risk of rheumatoid arthritis (1 day to 1 year) -SGLT2i vs. DPP-4i

|  | Patients with outcome | | Hazard ratio (95%CI) ^a^ |
| --- | --- | --- | --- |
|  | SGLT2i users  (n=206,069) | DPP-4i users  (n=206,069) |  |
| Rheumatoid arthritis | 802 | 916 | 0.914 (0.831-1.005) |
| Medical utilization |  |  |  |
| Hospitalization | 39961 | 46057 | **0.874 (0.863-0.886)^*^** |
| Emergency room visit | 29459 | 33637 | **0.899 (0.885-0.913)^*^** |
| Critical care | 6820 | 6932 | 1.018 (0.985-1.053) |
| All-cause mortality |  |  |  |
| Deceased | 3807 | 5123 | **0.776 (0.744-0.809)** |

Note:

SGLT2i: Sodium-glucose Cotransporter-2 Inhibitors, CI: Confidence interval.

If the patient is less or equal to 10, results show the count as 10.

a. Propensity score matching was performed on age at index, sex, race, socioeconomic status, DM severity, lifestyles, medical utilization, comorbidities, and HbA1C, eGFR, BMI, rheumatoid factor.

* Proportionality <0.001.

**Table S7.** Risk of rheumatoid arthritis (1 day to 1 year) -SGLT2i vs. Pioglitazone

|  | Patients with outcome | | Hazard ratio (95%CI) ^a^ |
| --- | --- | --- | --- |
|  | SGLT2i users  (n=80,846) | Pioglitazone  (n=80,846) |  |
| Rheumatoid arthritis | 344 | 368 | 0.922 (0.796-1.068) |
| Medical utilization |  |  |  |
| Hospitalization | 14057 | 16297 | **0.840 (0.821-0.859)** |
| Emergency room visit | 11059 | 12337 | **0.877 (0.854-0.899)** |
| Critical care | 2174 | 2402 | **0.893 (0.843-0.947)** |
| All-cause mortality |  |  |  |
| Deceased | 1182 | 1459 | **0.799 (0.740-0.863)** |

Note:

SGLT2i: Sodium-glucose Cotransporter-2 Inhibitors, CI: Confidence interval.

If the patient is less or equal to 10, results show the count as 10.

a. Propensity score matching was performed on age at index, sex, race, socioeconomic status, DM severity, lifestyles, medical utilization, comorbidities, and HbA1C, eGFR, BMI, rheumatoid factor.

* Proportionality <0.001.

**Table S8.** Risk of rheumatoid arthritis (1 day to 1 year)_stratified by sex

| Outcomes  (SGLT2i vs. Sulfonylureas users) | Hazard ratio (95%CI) ^a^ | |
| --- | --- | --- |
|  | Male  (n= 165,216 pairs) | Female  (n=132,474 pairs) |
| Rheumatoid arthritis | **0.881 (0.779-0.997)** | 0.924 (0.839-1.017) |
| Medical utilization |  |  |
| Hospitalization | **0.802 (0.790-0.813)** | **0.811 (0.798-0.824)^*^** |
| Emergency room visit | **0.835 (0.821-0.850)^*^** | **0.875 (0.859-0.892)** |
| Critical care | **0.846 (0.818-0.875)^*^** | **0.836 (0.801-0.873)^*^** |
| All-cause mortality |  |  |
| Deceased | **0.717 (0.686-0.750)** | **0.733 (0.692-0.777)** |

Note:

SGLT2i: Sodium-glucose Cotransporter-2 Inhibitors, CI: Confidence interval.

a. Propensity score matching was performed on age at index, sex, race, socioeconomic status, DM severity, lifestyles, medical utilization, comorbidities, and HbA1C, eGFR, BMI, rheumatoid factor.

* Proportionality <0.001.

**Table S9.** Risk of rheumatoid arthritis (1 day to 1 year)_stratified by age at index

| Outcomes  (SGLT2i vs. Sulfonylureas users) | Hazard ratio (95%CI) ^a^ | | |
| --- | --- | --- | --- |
|  | 20~44y  (n=33620 pairs) | 45~64y  (n=151079 pairs) | ≧ 65y  (n=125875 pairs) |
| Rheumatoid arthritis | 1.117 (0.818-1.526) | 0.926 (0.833-1.030) | **0.819 (0.742-0.905)** |
| Medical utilization |  |  |  |
| Hospitalization | **0.761 (0.732-0.790)** | **0.802 (0.789-0.816)^*^** | **0.836 (0.824-0.849)** |
| Emergency room visit | **0.845 (0.815-0.876)** | **0.847 (0.832-0.863)** | **0.893 (0.876-0.910)^*^** |
| Critical care | **0.885 (0.794-0.987)** | **0.829 (0.795-0.864)^*^** | **0.865 (0.835-0.896)^*^** |
| All-cause mortality |  |  |  |
| Deceased | **0.776 (0.618-0.974)** | **0.715 (0.669-0.764)** | **0.788 (0.755-0.823)** |

Note:

SGLT2i: Sodium-glucose Cotransporter-2 Inhibitors, CI: Confidence interval.

a. Propensity score matching was performed on age at index, sex, race, socioeconomic status, DM severity, lifestyles, medical utilization, comorbidities, and HbA1C, eGFR, BMI, rheumatoid factor.

* Proportionality <0.001.

**Table S10.** Risk of rheumatoid arthritis (1 day to 1 year)_stratified by race

| Outcomes  (SGLT2i vs. Sulfonylureas users) | Hazard ratio (95%CI) ^a^ | | |
| --- | --- | --- | --- |
|  | White  (n= 171,921 pairs) | Black/African American  (n= 41,621 pairs) | Asian  (n= 15,999 pairs) |
| Rheumatoid arthritis | 0.946 (0.858-1.042) | 1.002 (0.814-1.234) | 0.741 (0.488-1.124) |
| Medical utilization |  |  |  |
| Hospitalization | **0.834 (0.822-0.845)** | **0.817 (0.792-0.843)** | **0.787 (0.747-0.829)** |
| Emergency room visit | **0.855 (0.841-0.870)^*^** | **0.872 (0.845-0.900)** | **0.888 (0.833-0.947)** |
| Critical care | **0.833 (0.805-0.862)^*^** | **0.832 (0.774-0.895)** | **0.843 (0.756-0.940)** |
| All-cause mortality |  |  |  |
| Deceased | **0.747 (0.714-0.780)** | **0.675 (0.605-0.753)** | **0.691 (0.575-0.831)** |

Note:

SGLT2i: Sodium-glucose Cotransporter-2 Inhibitors, CI: Confidence interval.

a. Propensity score matching was performed on age at index, sex, race, socioeconomic status, DM severity, lifestyles, medical utilization, comorbidities, and HbA1C, eGFR, BMI, rheumatoid factor.

* Proportionality <0.001.

**Table S11.** Risk of rheumatoid arthritis (1 day to 1 year)_stratified by obese status

| Outcomes  (SGLT2i vs. Sulfonylureas users) | Hazard ratio (95%CI) ^a^ | |
| --- | --- | --- |
|  | With obesity ^b^  (n= 131,639 pairs) | Without obesity ^c^  (n= 134,026 pairs) |
| Rheumatoid arthritis | **0.867 (0.769-0.977)** | 0.919 (0.829-1.019) |
| Medical utilization |  |  |
| Hospitalization | **0.787 (0.775-0.800)^*^** | **0.844 (0.830-0.857)** |
| Emergency room visit | **0.862 (0.847-0.878)^*^** | **0.870 (0.853-0.888)** |
| Critical care | **0.810 (0.779-0.842)^*^** | **0.873 (0.841-0.906)** |
| All-cause mortality |  |  |
| Deceased | **0.676 (0.640-0.714)** | **0.782 (0.744-0.822)** |

Note:

SGLT2i: Sodium-glucose Cotransporter-2 Inhibitors, CI: Confidence interval.

a. Propensity score matching was performed on age at index, sex, race, socioeconomic status, DM severity, lifestyles, medical utilization, comorbidities, and HbA1C, eGFR, BMI, rheumatoid factor.
b. Diagnosed with overweight and obesity (ICD-10-CM code E66) or laboratory results of body mass index at least 30 kg/m^2^ within 1 year on or before the index date in both cohorts.
c. Never diagnosed with overweight and obesity (ICD-10-CM code E66) or laboratory results of body mass index at least 30 kg/m^2^ within 1 year on or before the index date in both cohorts.

* Proportionality <0.001.

**Table S12.** Risk of rheumatoid arthritis (1 day to 1 year)_stratified by HbA1C level

| Outcomes  (SGLT2i vs. Sulfonylureas users) | Hazard ratio (95%CI) ^a^ | |
| --- | --- | --- |
|  | **HbA1C < 7%^b^**  (n=27,856 pairs) | **HbA1C ≧ 9%^c^**  (n=64,105 pairs) |
| Rheumatoid arthritis | 0.959 (0.758-1.214) | 1.002 (0.806-1.245) |
| Medical utilization |  |  |
| Hospitalization | **0.756 (0.733-0.778)** | **0.832 (0.812-0.852)** |
| Emergency room visit | **0.864 (0.833-0.896)^*^** | **0.911 (0.888-0.935)** |
| Critical care | **0.736 (0.687-0.790)^*^** | **0.933 (0.875-0.995)** |
| All-cause mortality |  |  |
| Deceased | **0.628 (0.573-0.689)** | **0.811 (0.742-0.886)** |

Note:

SGLT2i: Sodium-glucose Cotransporter-2 Inhibitors, CI: Confidence interval.

a. Propensity score matching was performed on age at index, sex, race, socioeconomic status, DM severity, lifestyles, medical utilization, comorbidities, and HbA1C, eGFR, BMI, rheumatoid factor.
b. The results of laboratory test hemoglobin A1C was below 7%, and was not higher than 7% within 1 year on or before the index date in both cohorts.
c. The results of laboratory test hemoglobin A1C was higher than 9% within 1 year on or before the index date in both cohorts.

* Proportionality <0.001.

**Table S13.** Risk of rheumatoid arthritis (1 day to 1 year)_stratified by eGFR

| Outcomes  (SGLT2i vs. Sulfonylureas users) | Hazard ratio (95%CI) ^a^ | | |
| --- | --- | --- | --- |
|  | eGFR < 60 ml/min/1.73m^2^  (n=26,940 pairs) | eGFR 60~90 ml/min/1.73m^2^  (n=46,066 pairs) | eGFR > 90 ml/min/1.73m^2^  (n=46,036 pairs) |
| Rheumatoid arthritis | 0.931 (0.718-1.206) | **0.732 (0.582-0.920)** | 1.088 (0.863-1.371) |
| Medical utilization |  |  |  |
| Hospitalization | **0.798 (0.775-0.820)** | **0.803 (0.781-0.827)** | **0.811 (0.788-0.835)** |
| Emergency room visit | **0.879 (0.848-0.912)** | **0.897 (0.868-0.928)** | **0.872 (0.844-0.901)** |
| Critical care | **0.784 (0.734-0.838)** | **0.831 (0.768-0.900)** | **0.894 (0.823-0.971)** |
| All-cause mortality |  |  |  |
| Deceased | **0.762 (0.705-0.823)** | **0.681 (0.606-0.765)** | **0.674 (0.599-0.759)** |

Note:

SGLT2i: Sodium-glucose Cotransporter-2 Inhibitors, CI: Confidence interval. eGFR: estimated Glomerular filtration rate,

a. Propensity score matching was performed on age at index, sex, race, socioeconomic status, DM severity, lifestyles, medical utilization, comorbidities, and HbA1C, eGFR, BMI, rheumatoid factor.

* Proportionality <0.001.

**Table S14.** Risk of outcomes (1day to 1 year)_ propensity score matching with different variables

| Outcomes  (SGLT2i vs. Sulfonylureas users) | Hazard ratio (95%CI) | | | |
| --- | --- | --- | --- | --- |
|  | Model 1^a^ | Model 2^b^ | Model 3^c^ | Model 4^d^ |
| Rheumatoid arthritis | **0.840 (0.791-0.892)** | **0.904 (0.839-0.973)** | **0.925 (0.859-0.995)** | **0.899 (0.835-0.968)** |
| Medical utilization |  |  |  |  |
| Hospitalization | **0.920 (0.913-0.928)^*^** | **0.825 (0.816-0.833)^*^** | **0.881 (0.872-0.890)** | **0.815 (0.806-0.824) ^*^** |
| Emergency room visit | **0.946 (0.937-0.955)^*^** | **0.833 (0.823-0.843)^*^** | **0.911 (0.900-0.923)** | **0.869 (0.859-0.880)** |
| Critical care | **1.084 (1.062-1.107)^*^** | **0.907 (0.883-0.930)^*^** | **0.966 (0.941-0.991)^*^** | **0.847 (0.825-0.870)^*^** |
| All-cause mortality |  |  |  |  |
| Deceased | **0.845 (0.822-0.868)** | **0.806 (0.780-0.834)** | **0.835 (0.807-0.864)** | **0.740 (0.715-0.766)** |

Note:

SGLT2i: Sodium-glucose Cotransporter-2 Inhibitors, CI: Confidence interval.

a. Crude, before matching,
b. Propensity score matching was performed on age at index, sex, race, DM severity and HbA1C, rheumatoid factor.
c. Propensity score matching was performed on age at index, sex, race, socioeconomic status, DM severity, lifestyles, medical utilization, and HbA1C, rheumatoid factor.
d. Propensity score matching was performed on age at index, sex, race, socioeconomic status, DM severity, lifestyles, medical utilization, comorbidities, and HbA1C, eGFR, BMI, rheumatoid factor.

* Proportionality <0.001.

**Table S15.** Risk of rheumatoid arthritis (1 day to 1 year)_exclude switchers

|  | Patients with outcome | | Hazard ratio (95%CI) ^a^ |
| --- | --- | --- | --- |
|  | SGLT2i users  (n=206,549) | Sulfonylureas users  (n=206,549) |  |
| Rheumatoid arthritis | 880 | 899 | 0.933 (0.850-1.024) |
| Medical utilization |  |  |  |
| Hospitalization | 43412 | 53204 | **0.771 (0.761-0.781)^*^** |
| Emergency room visit | 31753 | 36311 | **0.824 (0.812-0.837)^*^** |
| Critical care | 7196 | 8332 | **0.830 (0.804-0.856)^*^** |
| All-cause mortality |  |  |  |
| Deceased | 4060 | 6226 | **0.622 (0.598-0.647)** |

Note:

SGLT2i: Sodium-glucose Cotransporter-2 Inhibitors, CI: Confidence interval.

If the patient is less or equal to 10, results show the count as 10.

a. Propensity score matching was performed on age at index, sex, race, socioeconomic status, DM severity, lifestyles, medical utilization, comorbidities, and HbA1C, eGFR, BMI, rheumatoid factor.

* Proportionality <0.001.

**Table S16.** Risk of rheumatoid arthritis (1 day to 1 year)_research network

|  | Patients with outcome | | Hazard ratio (95%CI) ^a^ |
| --- | --- | --- | --- |
|  | SGLT2i users  (n=336,946) | Sulfonylureas  users  (n=336,946) |  |
| Rheumatoid arthritis | 1321 | 1402 | **0.915 (0.849-0.987)** |
| Medical utilization |  |  |  |
| Hospitalization | 64538 | 76378 | **0.812 (0.804-0.821)^*^** |
| Emergency room visit | 43697 | 48286 | **0.873 (0.862-0.885)^*^** |
| Critical care | 9571 | 10556 | **0.884 (0.860-0.909)** |
| All-cause mortality |  |  |  |
| Deceased | 5524 | 7107 | **0.755 (0.729-0.782)** |

Note:

SGLT2i: Sodium-glucose Cotransporter-2 Inhibitors, CI: Confidence interval.

If the patient is less or equal to 10, results show the count as 10.

a. Propensity score matching was performed on age at index, sex, race, socioeconomic status, DM severity, lifestyles, medical utilization, comorbidities, and HbA1C, eGFR, BMI, rheumatoid factor.

* Proportionality <0.001.

**Table S17.** Risk of rheumatoid arthritis (SGLT2i vs. Sulfonylureas users)

| Follow up | Patients with outcome | | Hazard ratio (95%CI) ^a^ |
| --- | --- | --- | --- |
|  | SGLT2i users  (n=310507) | Sulfonylureas  users  (n=310507) |  |
| 1 day to 1 year | 1343 | 1454 | **0.899 (0.835-0.968)** |
| 1 day to 3 years | 2433 | 2470 | 0.950 (0.899-1.005) |
| 1 day to 5 years | 2888 | 2861 | 0.972 (0.923-1.023) |
| 1 day to 7 years | 3045 | 2993 | 0.976 (0.928-1.026) |

Note:

SGLT2i: Sodium-glucose Cotransporter-2 Inhibitors, CI: Confidence interval.

a. Propensity score matching was performed on age at index, sex, race, socioeconomic status, DM severity, lifestyles, medical utilization, comorbidities, and HbA1C, eGFR, BMI, rheumatoid factor.

# 2 Supplementary Figures

**Figure S1**. Selection process (SGLT2i vs. DPP-4i)

T2DM = type 2 diabetes mellitus; SGLT2i: Sodium-glucose Cotransporter-2 Inhibitors; DPP-4i = Dipeptidyl peptidase 4 inhibitors; SES = socioeconomic status; HbA1c = hemoglobin A1C; eGFR = estimated glomerular filtration rate; BMI = body mass index.

**Figure S2.** Selection process (SGLT2i vs. Pioglitazone)

T2DM = type 2 diabetes mellitus; SGLT2i: Sodium-glucose Cotransporter-2 Inhibitors; SES = socioeconomic status; HbA1c = hemoglobin A1C; eGFR = estimated glomerular filtration rate; BMI = body mass index.
